# Regulated cell death: discovery, features and implications for neurodegenerative diseases

**DOI:** 10.1186/s12964-021-00799-8

**Published:** 2021-12-18

**Authors:** Juntao Cui, Suhan Zhao, Yinghui Li, Danyang Zhang, Bingjing Wang, Junxia Xie, Jun Wang

**Affiliations:** 1 School of Basic Medicine, Qingdao University, Qingdao, 266071 China; 2 Institute of Brain Science and Disease, Shandong Provincial Key Laboratory of Pathogenesis and Prevention of Neurological Disorders, Qingdao University, Qingdao, 266071 China; 3 School of Clinical Medicine, Qingdao University, Qingdao, 266071 China

**Keywords:** Apoptosis, Pyroptosis, Autophagy-dependent cell death, Necroptosis, Ferroptosis

## Abstract

Regulated cell death (RCD) is a ubiquitous process in living organisms that is essential for tissue homeostasis or to restore biological balance under stress. Over the decades, various forms of RCD have been reported and are increasingly being found to involve in human pathologies and clinical outcomes. We focus on five high-profile forms of RCD, including apoptosis, pyroptosis, autophagy-dependent cell death, necroptosis and ferroptosis. Cumulative evidence supports that not only they have different features and various pathways, but also there are extensive cross-talks between modes of cell death. As the understanding of RCD pathway in evolution, development, physiology and disease continues to improve. Here we review an updated classification of RCD on the discovery and features of processes. The prominent focus will be placed on key mechanisms of RCD and its critical role in neurodegenerative disease.

**Video abstract**

**Supplementary Information:**

The online version contains supplementary material available at 10.1186/s12964-021-00799-8.

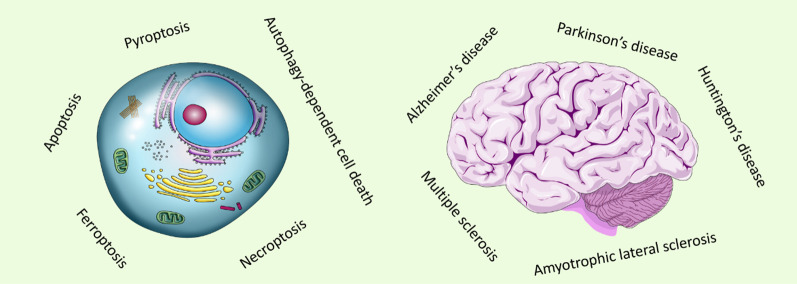

## Background

Cell death has been viewed as unavoidable consequence of cellular life. Cells may be dead by accidental cell death (ACD) or regulated cell death (RCD). ACD is an accidental and uncontrolled biological process, however RCD is implicated in closely integrated signaling cascades and molecular-mediated effector mechanisms. The deep comprehension of these lethal subroutines and their interplay outcomes may reveal novel therapeutic targets for the reduction of pathological cell loss and excessive cell proliferation [1]. The completely physiological form of RCD is also known as programmed cell death (PCD) [2]. Neurodegenerative diseases are a variety of pathologies, such as Alzheimer’s disease (AD), Parkinson’s disease (PD), Amyotrophic lateral sclerosis (ALS), Multiple sclerosis (MS) and Huntington’s disease (HD), resulting in the progressive degeneration of the nervous system, including widespread neuronal cell death [3–7]. Currently, there are several hallmarks for the pathology of neurodegenerative diseases. One is the progressive loss of selectively vulnerable populations of neurons, another is microglia-mediated neuroinflammation [8, 9]. The third is deposition of protein aggregates with abnormal conformational properties including amyloid β-protein (Aβ), tau, and α-synuclein [10, 11]. Accumulating evidence shows that RCD is highly related in neurodegenerative diseases. Here, we critically discuss the current knowledge of RCD, including apoptosis, pyroptosis, autophagy-dependent cell death, necroptosis, ferroptosis. We hope through concluding and comparing these cell death types to explore what the linkage between them, how mechanisms execute neuronal cell death and how to regulate cell survival or death and further to cure diseases. Understanding of such cell death provides novel insights into the pathogenesis of human diseases and establishment of preventive and therapeutic strategies.

## Apoptosis

### The definition and discovery of apoptosis

Apoptosis is a form of PCD regulated by genes under physiological or pathological stimuli. The key of the apoptotic machinery is conserved. It is an initiative mode of cell death caused by stimuli factors in both vivo and vitro to trigger the pre-stored cell death program, which is different from necrosis in morphology and biochemical characteristics. Necrosis is instantaneous, catastrophic, and cannot be prevented through pharmacological or genetic interventions [2]. In 1972, Kerr et al. termed apoptosis as a form of PCD with morphology distinct from necrosis. This process depended on the activation of caspase family, without the rupture of mitochondria, lysosomes and cell membranes, and without the overflow of contents, so there was no inflammatory reaction [12]. Caspases are an evolutionarily conserved family of cysteine proteases existed as inactive zymogens in almost all cells. It is confirmed that apoptotic caspases mediate apoptosis, including caspase-2, caspase-3, caspase-6, caspase-7, caspase-8, and caspase-9. In addition, the exclusive caspase-10 to human being [13, 14]. The loss of active death signals or survival signals cause apoptosis. When the pro-death signal exceeds the pro-survival signal, cell apoptosis is triggered. Apoptosis was triggered by both intrinsic stimulus, such as DNA damage, growth factor withdrawal, endoplasmic reticulum stress, reactive oxygen species (ROS) overload, and extrinsic stimulus, such as steroid hormones or ligation of death receptors, resulting in the activation of caspases [15]. Despite apoptosis is generally referred to as an immunologically silent process without inflammatory response, new evidence demonstrate that apoptosis can be inflammatory under certain conditions and play a significant role in the host defense against infection [16]. In mammals, there are two classic apoptosis pathways, extrinsic apoptosis pathway and intrinsic apoptosis pathway. In 2018, Nomenclature Committee on Cell Death (NCCD) proposes to define intrinsic apoptosis as a form of RCD initiated by perturbations of the intracellular or extracellular microenvironment, demarcated by mitochondrial outer membrane permeabilization (MOMP) and executed by executioner caspases, mainly caspase-3. Extrinsic apoptosis define as a specific variant of RCD, initiated by perturbations of the extracellular microenvironment detected by plasma membrane receptors, propagated by caspases-8 and mainly precipitated by executioner caspases-3 [2].

### The features and pathway of apoptosis

Apoptosis is a stochastic and classical PCD. The representative morphological features are cell shrunk, condensed chromatin, DNA fragmentation, mitochondrial swelling. Nuclei are cleaved into fragments and cells are divided into several apoptotic bodies with membrane wrapped by the cell membrane [13, 17]. It is now confirmed that apoptotic cells release many signals that profoundly impact their cellular environment rather than the traditional notion that dying cells were thought to be rapidly cleared by phagocytes with limited signaling capacity [18]. Under the influence of the metabolic changes of apoptotic cells and the activity of apoptosis-related channel proteins, different types of cell can release similar metabolites after apoptosis [19]. Phosphatidylserine phosphatidylserine (PtdSer) exposure is considered to be a hallmark of apoptosis, and Annexin V is a detect marker of apoptotic cells which can specifically bind to PtdSer on the cell membrane. However, the presence of Annexin V just indicates phosphatidylserine exposure which is insufficient to distinguish specific cell death types with cell membrane intact. In addition, the terminal deoxynucleotidyl transferase-mediated dUTP nick-end labelling (TUNEL) is access for detecting apoptotic cells in research, because apoptosis is accompanied by the cleavage of chromosomal DNA into nucleosomal units [20, 21]. Apoptotic caspases cleave cellular substrates, leading to the condensation of cytoplasm, chromatin condensation, DNA cleavage and maintenance of intact plasma membrane in morphology [22]. In vivo, without inflammation of PtdSer signal recognized by phagocytes, this process attracts phagocytes to engulf apoptotic cells without evoking inflammatory responses [23, 24].

In general, caspases have two categories, initiator caspases and executioner caspases. The initiator caspase of apoptosis consists of caspase-2/8/9/10/12, and the executioner caspases include caspase-3/6/7 and-14 [13, 25]. The functions of both caspases-2 and -14 have not been fully clear. It is reported that caspase-12 is phylogenetically associated with the inflammatory caspase subfamily and the current consensus is that caspase-12 is dispensable for apoptosis [13, 26]. Initiator caspases with long amino-terminal pro-domains promote the formation of the apoptosome, which is considered to initiate apoptosis. The apoptosome consists of caspase-9 pro-domain with apoptotic protease-activating factor 1 (APAF1) and cytochrome C and mediates the formation of caspase-9 homodimers and capase-9 with APAF1 herterodimers. Caspases-3 and -7 regulate downstream events such as extensive MOMP and the release of cytochrome C. MOMP, which causes the release of cytochrome C from the mitochondrion, is a key point to initiate cell death in the intrinsic apoptosis pathway [27, 28]. MOMP is controlled by members of the BCL-2 family, containing with both anti-apoptotic and pro-apoptotic members. The interaction between BCL-2 proteins determines whether the cell commits to apoptosis [2].

**Fig. 1:**
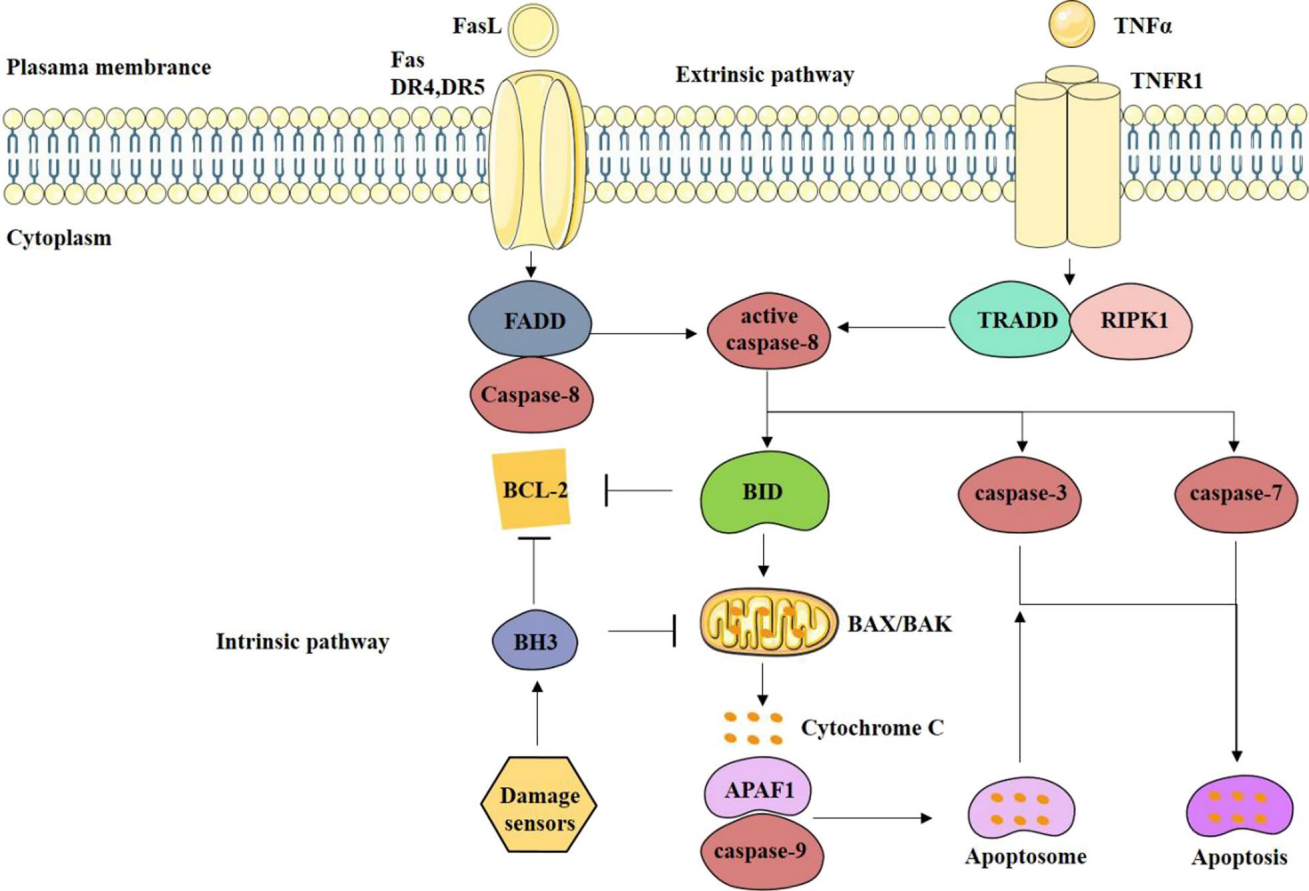
Overview of extrinsic and intrinsic pathway of apoptosis. External way of apoptosis is mediated by death receptor (DR). DR is activated by binding to the corresponding ligand, and is activated by the initial caspase (caspase-8) and the effector caspase (caspase-3/7), which eventually leads to cell apoptosis. After FasL binds to DR Fas, DR4 or DR5, it causes local oligomerization and activation of Fas molecules. Fas recruits Fas-related death domain protein (FADD) in its cytoplasm activated Caspases-8 and triggerd apoptosis. Tumor necrosis factor (TNF) receptor 1 (TNFR1) ligation recruits early complexes composed of TNFR1-associated death domain protein (TRADD) and receptor-interacting serine/threonine protein kinase 1 (RIPK1) and recruits caspase-8 and makes dimerization. Active caspase-8 cleaves downstream targets to activate two different pathways: directly cleaves the executioner caspases, caspase-3 and caspase-7, or engages the cell-intrinsic pathway to amplify executor caspase by processing BH3-only protein BID activation. Caspases-3 and Caspases-7 also regulate the permeability of the outer membrane permeabilization and the release of cytochrome C. In the intrinsic pathway, multiple stimuli that cause cellular stress or damage usually activate one or more members of the BH3-only protein family. BH3-only protein activation exceeding a critical threshold overcomes the inhibitory effect of anti-apoptotic B-cell lymphoma-2 (BCL-2) family members and promotes the assembly of BAK-BAX oligomers in the outer mitochondrial membrane. Activated BH3 protein activates BCL-2 antagonist/killer (BAK) and BCL-2 associated X protein (BAX) to induce mitochondrial outer membrane permeability and cytochrome C release. Cytochrome C binds and oligomerizes apoptotic protease-activating factor 1 (APAF1), which recruits and activates caspase-9. Cytochrome C and APAF1 combine to form an apoptosome that drive the activation of caspase-9, which stimulates caspase-3 and -7, and then induces apoptosis

As shown in Fig. 1 extrinsic apoptosis pathway also called death receptor-mediated apoptosis pathway. The death receptor is initiated via binding to the corresponding ligand. According to different downstream cascade reactions, it can be divided into two categories. One type of apoptosis cascade should be mediated by the death receptor FAS (also called CD95 or APO-1). Fas recruits Fas-related death domain protein (FADD) in its cytoplasm activated Caspases-8 and triggerd apoptosis, the formation of the death signal-induced complex is a key step in the cascade. Another type is that the apoptosis cascade is initiated by the death receptor, Tumor necrosis factor (TNF) receptor 1 (TNFR1) ligation recruits early complexes composed of TNFR1-associated death domain protein (TRADD) and receptor-interacting serine/threonine protein kinase 1 (RIPK1). After a series of downstream signal cascades, initial caspase (caspase-8) and effector caspase (caspases-3/7) are activated step by step, which finally leads to apoptosis [2]. During intrinsic apoptosis pathway, BCL-2 proteins are pivotal regulators. Each member of the BCL-2 family composes more than one BCL-2 homology (BH) domains, BH1, BH2, BH3, and/or BH4 [29, 30]. The activation of BAX or BAK at the mitochondrial surface are activated by BH3-only proteins resulting in an allosteric change, subsequently, which can enable them to oligomerize and form macropores in the membrane, contributing to MOMP and the release of cytochrome C. Cytochrome C released into the cytoplasm binds to APAF-1 and caspase-9, leading to the activation of caspase-9 which subsequently activates caspase-3 and causes apoptosis. Active caspase-8 cleaves downstream targets to activate two different pathways: directly cleaves the executioner caspase-3 and caspase-7 or catalyzes the cleavage of BID (the pro-apoptotic molecule of BCL-2) into two fragments, in which the C-terminal fragment containing the BH3 domain is transported to the mitochondria causing high-efficiency release of cytochrome C from the mitochondria and then induces apoptosis [2, 31]. The proportion of BCL-2 family members is one of the core mechanisms of pro-apoptosis and inhibitory apoptosis pathways especially the BCL-2/BAX ratio, which is a molecular switch for initiating apoptosis. BAX and BCL-2 regulated apoptosis by forming homologous or heterodimer: BAX induced apoptosis when forming homologous dimer. When BAX and BCL-2 formed heterodimer, apoptosis was inhibited [32, 33]. In the present study, a simplified extrinsic and intrinsic pathway of apoptosis definition were summarized (Fig. 1).

### Apoptosis and neurodegenerative diseases

More and more evidence has confirmed that in human diseases, deregulated apoptosis is implicated in the pathological loss or accumulation of cells [34, 35]. In physiology, apoptosis can remove damaged or redundant cells to ensure organism homeostasis. Excessive apoptosis may be deleterious too, such as neuronal cell death in neurodegenerative diseases. AD is a devastative neurodegenerative disorder with complex etiology. Neuronal apoptosis is an important component of AD because neuronal apoptosis is found early in AD, and a large number of apoptotic neurons in the cerebral cortex and hippocampus have also been found in autopsies of AD patients [36, 37]. Current treatments for AD include acetylcholinesterase inhibitors such as donepezil, rivastigmine, and galantamine that can be used to treat mild to moderate AD, and memantine, an antagonist of the glutamate receptor *N*-methyl d-aspartate (NMDA) subtype, is an alternative treatment for severe AD [38, 39]. However, these drugs provide only short-term symptomatic improvement and do not alter the progression of the disease. PD is a progressive neurodegenerative disease. Typical motor symptoms include resting tremor, bradykinesia, rigidity, and postural instability, accompanied by loss of dopaminergic neurons and lewy pathology [5, 40]. To date, at least three genes have been found to be relevant with PD. Parkin deficiency, which is a main pathogenesis of familial PD, may contribute to elevate apoptotic sensitivity of cultured neural lineage cells. Accumulating evidence in human and various animal models of PD indicate that mitochondrial dysfunction plays an important role in early PD pathogenesis and is likely to be a shared feature between sporadic and monogenic form of PD [41, 42]. In PD mouse models, p53-mediated upregulation of BAX is a critical progress in the substantia nigra pars compacta (SNpc) dopaminergic neuron apoptosis caused by mitochondrial dysfunction (inhibition of respiratory complex I) [43]. An increase in the number of mitochondrial damages can trigger apoptosis through intrinsic pathways and ROS generation, which oxidizes membrane lipids and destroys the stability of lysosomal membranes. Moreover, many studies have suggested that the autophagy-lysosome system has been found to be damaged in postmortem PD patient tissue and PD models [44, 45]. Autophagy may promote cell apoptosis by reducing the clearance of other pro-apoptotic factors, such as activated caspases. Thus, an imbalance between autophagy and apoptosis may be a cause of PD.

ALS is a progressive neurodegenerative disease for which the pathophysiological mechanisms of motor neuron loss are not precisely clarified [4]. There is increasing evidence that a programmed mechanism of cell death resembling apoptosis is responsible for motor-neuron degeneration in ALS. MAP4K4 as a key regulator of motor neuron degeneration in ALS, blocking MAP4K4 attenuated JNK3-cJun-induced motor neuron apoptosis [46]. SOD1^G93A^ mouse is the most widely used fALS model [47, 48]. This mouse reduces the folding stability of SOD1 and induces the formation of protein aggregates. In SOD1^G93A^ mouse model, the level of anti-apoptotic BCL-2 decreased abnormally and the expression of apoptosis effector BAX in spinal cord motor neurons of ALS patients increased [49–51]. Activation of RIPK1-mediated neuroinflammation and cell death is directly linked with the ALS. Inhibition RIPK1 protects against the degeneration of oligodendrocytes in SOD1^G93A^ transgenic mice, which occur before the onset of motor dysfunction. This suggest that RIPK1 might promote axonal degeneration and neuroinflammation noncell autonomously in ALS [52, 53]. HD is an autosomal-dominant, progressive neurodegenerative disease with the clinical symptoms of chorea and dystonia, incoordination, cognitive decline, and behavioral abnormal which is characterized by the presence of the aggregated mutant huntingtin (mHTT) protein [54–56]. Over the years, a large number of studies have shown that anomalous apoptosis plays a role in the pathology of HD. Apoptotic cells and DNA degradation products were observed in the brain of HD patients and experimental HD models [57–61]. 3-Nitropropionic acid (3-NP) is an irreversible succinate dehydrogenase inhibitor and also a naturally synthesized plant mycotoxin which produces selective injuries in striatum in both experimental animals and in humans mimics the effects of HD. A recent study indicate that dapagliflozin improves behavioral dysfunction of HD in rats via inhibiting apoptosis-related glycolysis, its demonstrated anti-apoptotic, anti-glycolytic, anti-inflammatory and autophagic effects on 3-NP-damaged striatal cells [62]. MS is a disseminated chronic inflammatory demyelinating disease of the central nervous system (CNS). It is the most common neurological disease in young people, accompanied by progressive axonal degeneration. The pathogenesis of MS is far from being elucidated. However, there is increasing evidence that inflammation and apoptosis may play a role at the patients peripheral level or in the CNS [63, 64]. The expression of Mcl-1 protein activated monocytes in MS patients was up-regulated, and the expression of pro-apoptotic Bak in recurrent MS patients was decreased, which confirmed the trend of PBMC resistance to apoptosis in MS patients [65]. IAP family proteins are key regulators of apoptosis. A lack of response to apoptosis triggering events was observed in peripheral blood and encephalitis T cells of experimental allergic encephalomyelitis (EAE) mice (a disease model of MS) which was related to the up regulation of XIAP protein expression [66]. Peripheral blood lymphocytes of MS patients show the characteristics of activated cells, and the imbalance of apoptosis may further aggravate this phenomenon. Taken together, although we still do not know whether apoptosis is the cause or consequence of neurodegenerative processes, but, more and more evidence shows that apoptosis plays a central role in several neurodegenerative diseases. This research field may still bring promising results and constructive treatment options for patients with neurodegenerative diseases.

## Pyroptosis

### The definition and discovery of pyroptosis

Pyroptosis is a novel type of programmed cell necrosis. Different from immune silencing apoptosis, pyroptosis is a form of lytic inflammatory cell death mediated by inflammatory caspases (caspase-1/4/5 in human and murine caspase-11). The morphology of pyroptosis is cell swelling and membrane rupture [16]. Initially, pyroptosis is regarded as the caspase-1 dependent programmed cell death. Brennan and Cookson have discovered the cell death induced by *Salmonella typhimurium* and largely distinct from a classical apoptotic mechanism at first. They found that a novel caspase-1-dependent mechanism of necrosis killed *Salmonella*-infected macrophages [67, 68]. During pyroptosis, many cytokines and danger signal molecules are released in pyroptosis, which activates the immune system and leads to inflammatory response. Pyroptosis is initiated by a range of microbial infections, such as *Salmonella* and *Legionella*, and non-infectious stimuli, such as host factors produced during myocardial infarction [68]. Gasdermin-D (GSDMD)-mediated pyroptosis is an important innate immune response to antagonize pathogen infection, but excessive response can cause a series of diseases including sepsis [69].

In 1992, Sansonetti and his colleagues reported that *Shigella flexneri* can cause macrophage death after infection entered the cell in nature [70]. With the use of electron microscopy, they found that this type of cell death was chromatin condensation, blebbing of the cell membrane, cytoplasmic void bubble formation, endoplasmic reticulum swelling, and organelle structure is still retained. Afterward, through electrophoresis of the genome, the study observed DNA fragmentation in pyroptosis which is similar to apoptosis. By 1994, study further analyzed and found that macrophages could release a large amount of IL-1 after *Shigella flexneri* was infected with pyroptosis, but IL-6 and TNF-α were not observed [71]. In 1996, Chen et al. d that caspase-1 was activated in this type of cell death, and caspase-1 inhibitors can suppress cell death. It is the primarily report that caspase-1 can cause cell death [72]. Previous studies showed that activated caspase-1 mediates proteolytic cleavage of the inflammatory precursor cytokines pro-IL-1β and pro-IL-18 [73, 74]. These studies together provided important evidences for ensuring a new form of cell death, but some people thought it as a novel apoptosis with inflammation. In 2001, Cookson and Brennan originally proposed the term 'pyroptosis' to define a special type of RCD like apoptosis to some extend but dependent on inflammatory reaction and caspase-1 [68]. Furthermore, in 2018, the NCCD refers to pyroptosis as a form of RCD that critically depends on the formation of plasma membrane pores by members of the gasdermin protein family, often (but not always) as a consequence of inflammatory caspase activation [2].

### The features and pathway of pyroptosis

Pyroptosis is lytic, proinflammatory with production of activated inflammatory cytokines, as well as rapidly plasma membrane rupture and release of inflammatory intracellular contents [67]. Inflammatory caspases (caspase-1/4/5 and-11) are critical in pyroptosis, because they activate the proinflammatory cytokines IL-1β and IL-18 [72]. Pyroptosis is dependent on gasdermin protein, executioner of cell death [67, 75]. Inflammasome can activate GSDMD to drive pyroptosis via forming membrane pores and releasing inflammatory response [76, 77]. Inflammasomes are divided into canonical and noncanonical, depending upon which caspase is engaged in activation [78]. Both canonical and noncanonical inflammasomes can directly mediate GSDMD cleavage. Pore-forming protein GSDMD is the executioner of pyroptosis, which is cleaved by inflammatory caspases and determines whether pyroptotic cell death or not [76]. Canonical inflammasome activation activate caspase-1, which has a high affinity for GSDMD and pro-forms of IL-1β and IL-18. Noncanonical inflammasomes activate human caspase-4/5 or mouse homologue caspase-11 to drive pyroptosis. The inflammasome consists of a sensor protein, a caspase-1 family protease and apoptosis-associated speck-like protein containing C-terminal caspase recruitment domain (CARD, also called ASC). To date, there are five typical inflammasome sensors, including NOD-like receptor (NLR) family pyrin domain-containing 1 (NLRP1), NLRP3, NLR family caspase recruitment domain-containing 4, absent in melanoma 2, and Pyrin [79]. Inflammasomes are critically implicated in components of the innate immune system, which is the first line of host defense following infectious and sterile insults. These macromolecular complex assemble is initiated by sensor molecule in response to diverse stimuli [80].

**Fig. 2:**
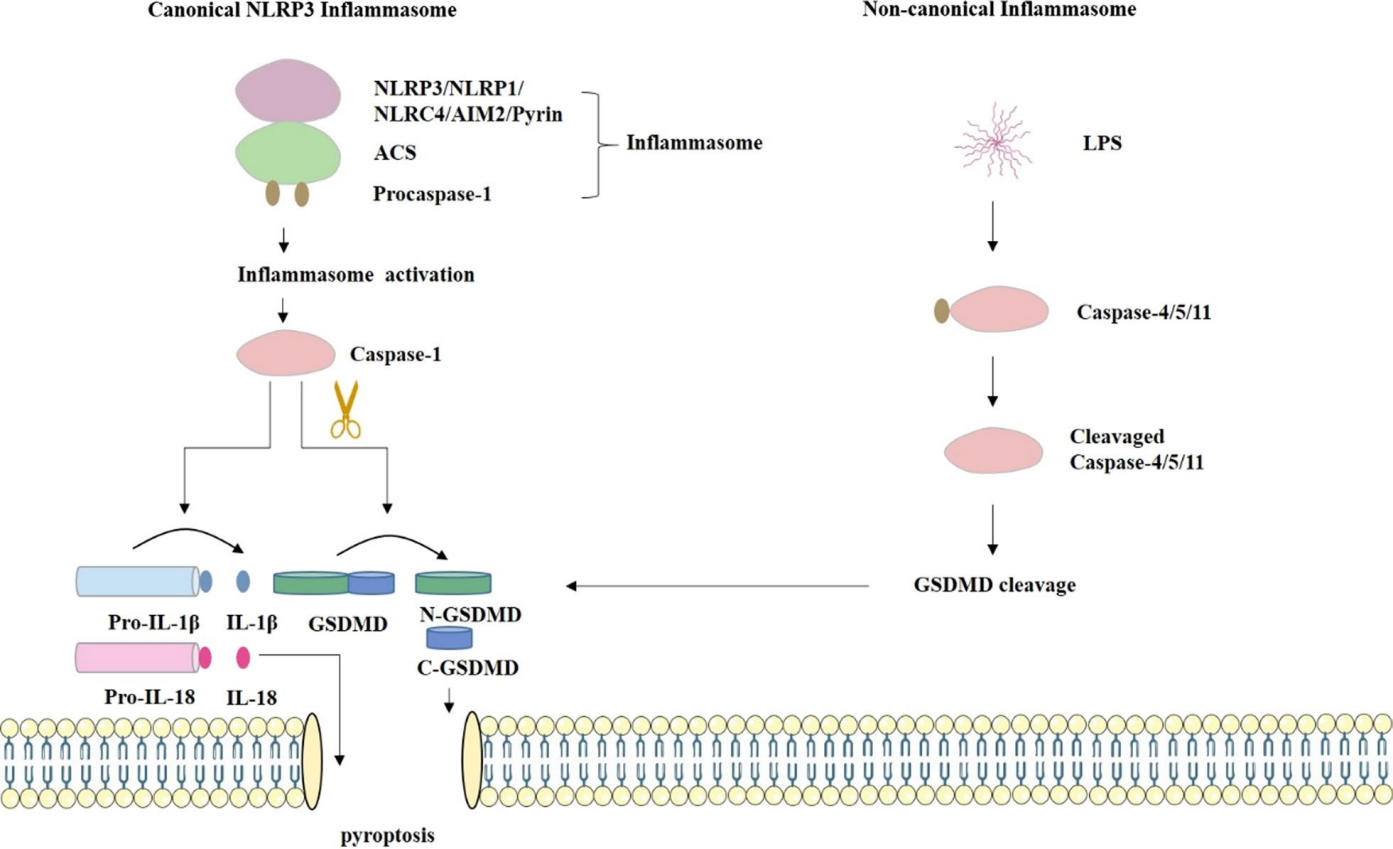
Molecular mechanism of pyroptosis. Caspase-1-dependent pyroptosis requires activation of the canonical inflammasomes. In this pathway, pathogen-associated molecular patterns activate their respective inflammasome sensors, including NLRP3, NLRP1, NLRC4, AIM2, and Pyrin. These inflammasomes recruit ASC adaptors, and the NLR or AIM2 signaling domains are connected to the ASC through homotypic interactions which generates the formation of ASC focus. The ASC focus recruits procaspase-1, leading to the activation of caspase-1. Noncanonical inflammasome direct recognition of the cytosolic lipopolysaccharide (LPS), which is derived from gram negative bacteria and can directly bind to and active caspase-4/5/11. GSDMD is the direct substrate of caspase-1/4/5/11, which can be specifically cleaved by inflammatory caspase and plays an important role in the downstream of inflammatory caspase. GSDMD exists in an autoinhibitory conformation at homeostasis, in which the inhibitory C terminal domain (C-GSDMD) retains the pore-forming N terminal domain (N-GSDMD) in an inactive state. Upon processing by the activated caspases, the GSDMD N terminal is released and translocated to the inner plasma membrane. Activated caspase-1 also cleave and activate the proinflammatory cytokines interleukin (IL)-1β and IL-18, which are released through GSDMD pores

As shown in Fig. 2, there are two pathways, the canonical and non-canonical pathways. In the canonical pathway, stimuli associated with pathogens or released from dying cells trigger the formation of inflammasomes, which are multiprotein complexes for the processing and activation of caspase-1. There exists an alternative way for activation of the NLRP3 inflammasome termed as non-canonical NLRP3 inflammasome pathway. Mouse caspase-11, or the human analogues caspase-4/5, upon gram-negative bacteria infection, directly bind to the bacterial cell wall component LPS and subsequently cleave GSDMD to drive NLRP3 inflammasome initiation [80]. Therefore, in this process, LPS sensed by caspase-11 (or caspase-4/5) performs as the upstream to trigger the assemble of NLRP3 inflammasome. Inflammasome sensors interact with the adapter molecule ASC in the cytosol, then recruit and activate caspase-1 [77]. In both the canonical and non-canonical pathways of pyroptosis, caspase-1/4 /5/11 specifically cleaves an executor protein called GSDMD, and transfroms pro-IL-1β and pro-IL-18 into their mature forms. Then the approximately 30 kDa amino-terminal domain of GSDMD translocate to the plasma membrane and forms pores to cause pyroptosis and the release of the processed mature forms of IL-1β and IL-18 [81, 82]. GSDMD membrane pore formation is key step in the mechanism of pyroptosis. Thus, inflammasomes play an important role in inflammation via the release of IL-1β and IL-18. The interactions between canonical and noncanonical inflammasome pathways may co-promote the inflammatory response and drive pyroptosis. In the present study, a simplified molecular mechanism of pyroptosis definition were summarized (Fig. 2).

### Pyroptosis and neurodegenerative diseases

Currently, pyroptosis has been proved to be related with pathogenesis of inflammatory, metabolic diseases, neurodegenerative diseases [16, 81, 83, 84]. Inflammasome is a multiprotein complex involved in the innate immune system, causing inflammatory responses and pyroptosis. Among the various types of inflammasomes, NLRP3 inflammasome is the well-known in neurodegenerative diseases, especially in AD and PD and the activation of the NLRP3 inflammasome causes the production of IL-1β and IL-18 in microglia cells [81, 85]. Substantial evidence supports a role for inflammasomes in the pathogenesis of AD, a devastative neurodegenerative disorder with complex etiology. The inflammatory process is considered to contribute to the neurodegeneration of AD. Microglial-derived proinflammatory cytokines is a key that mediates neuronal loss and maintains microglia activation, leading to further cell damage in AD with IL-1β and -18 exacerbating the disease [86, 87]. This was shown by the detection of IL-18 in microglia, astrocytes, and neurons in the AD brain. Similarly, in the brains of AD patients IL-1β Sustained expression, particularly in microglia and astrocytes, and also detectable in cerebrospinal fluid [86, 88–90]. Compared with wild type mice, NLRP3 and caspase-1 KO mice both demonstrate improved memory, accompanied by an anti-inflammatory microglial profile [91]. Therefore, the inflammasome plays a crucial role in regulating neuroinflammation which may be a vital therapeutic molecular target for AD. Recent studies have shown that inflammatory corpuscle activation is also related to PD, inhibite inflammatory inflammasome pathway can prevent the death of dopaminergic neurons [92, 93]. The activation of NLRP3 inflammasome in microglial is highly relevant with dopaminergic neuronal loss and subsequent motor dysfunction in the 1-methyl-4-phenyl-1,2,3,6-tetrahydropyridine (MPTP)-treated mouse, which is a common PD animal model. NLRP3 deficiency profoundly relieves motor dysfunctions and dopaminergic neurodegeneration in MPTP-treated mice [94]. Cleaved caspase-1 and the inflammasome adaptor protein ASC can be observed increased in the substantia nigra of PD patients and multiple PD models. Nanomolar doses of a small-molecule NLRP3 inflammasome inhibitor, MCC950, eliminates fibrillar α-synuclein-mediated inflammasome activation in mouse microglial cells and extracellular ASC release [92]. A recent study showed evidence that rotenone activates NLRP3 inflammasome and induces pyroptosis. NIM811 protects cells from rotenone-induced damage and inhibits NLRP3 inflammasome and pyroptosis. This suggests that NIM811 might serve as a potential therapeutic drug for PD [95]. Interestingly, accumulating evidences indicate that autophagy in microglial is involved in the neuroinflammation [96]. The autophagy inducers, such as rapamycin, metformin, and AICA Riboside, can effectively prevent the excessive activation of the NLRP3 inflammasome. NLRP3 inflammasome can impairs microglial autophagy, suggesting that NLRP3 inflammation inhibition is a promising therapeutic strategy for PD [93, 97, 98].

Numerous studies have demonstrated elevated expression of IL-18, IL-1β, NLRP3, and caspase-1 in the serum, CSF, and leukocytes of patients with active MS, and CSF IL-1β levels correlate with the number and volume of cortical demyelinating lesions, as well as the severity of the disease course [99–103]. In EAE, genetic deletion of IL-1β, NLRP3, ASC, pyrin, caspase-11 or GSDMD can reduced the neuroinflammation and disease severity. Pharmacological intervention has always supported the pathogenic role of inflammatory inflammasome in EAE. NLRP3 inflammasome inhibitor MCC950 decreases IL-1β production in vivo and attenuates the severity of EAE. When administered at EAE onset, the caspase-1 inhibitor VX-765 blocked pyroptosis, reduced neuroinflammation, and prevented neurodegeneration [104–106]. Similarly, GSDMD inhibitor disulfiram also can attenuate the course of EAE [107]. Unlike AD and PD, the presence of peripheral immune cells in the brain is not a typical finding in HD. Several oxidative stress and inflammation markers including CRP, GM-CSF, TNF, IL-1β, IL-6 and IL-8 were observed to be elevated in the serum of HD patients [108–112]. A recent study in striatal neurons of R6/2 mouse model of HD showed that NLRP3 and caspase-1 were strongly expressed in 13 week old R6/2 mice. At the same time, NLRP3 is highly expressed in striatal spiny projection neurons and in parvalbumin interneurons, which are prone to degenerate in HD [113]. The origin of neuroinflammation and whether inflammation suppression can effectively reduce the progression of this disease, it will be interesting to explore NLRP3 suppression or the use of other immunosuppressive agents. Neuroinflammation is considered to be an important factor in the progression of ALS [114–116]. Inflammation induced neurotoxicity leads to the activation of microglia and astrocytes to produce IL-1 β, Further lead to motor neuron death [48, 117]. The high levels of caspase-1 and IL-1β in microglia contribute to disease progression in the mouse SOD1^G93A^ model, indicating the role of microglia NLRP3 in ALS. LPS activates caspase-1, leading to increased IL-1β release in SOD1^G93A^ mice [117, 118]. In ALS patients and ALS mouse models, NLRP3 and its inflammatory components caspase-1 and IL-1 β. It is activated and up-regulated, indicating that NLRP3 complex plays a key role in ALS pathology [117, 119]. As inflammatory signaling hubs in the CNS, inflammasomes are key mediators in the involvement between inflammation and cell death in the CNS. Thus, understanding how these molecules activate pyroptosis and how inflammasomes activation or IL-18/IL-1β maturation ultimately leads to this unique form of PCD have implications for not only understanding bacterial pathogenesis, but also a better understanding of several neurodegenerative diseases.

## Autophagy-dependent cell death

### The definition and discovery of autophagy-dependent cell death

Autophagy is a conserved catabolic process that refers to as a self-sacrificing mechanism to degrade cellular contents and recycle damaged organelles. Autophagy can contribute to survival or death, therefore autophagy plays an important role in cell fate and the maintenance of cell metabolic balance [120]. The process of autophagy consists of the subsequent formation of four unique membrane structures, namely phagophore, autophagosome, lysosome and autolysosome. Autophagy is a process of self-eating through forming a dedicated engulfing double-membrane vesicle called autophagosome and degradation of proteins and organelle inside the lysosome [25]. There are three major autophagy types: macroautophagy, microautophagy, and chaperone mediated autophagy (CMA). According to the selectivity of substrate degradation, autophagy can be divided into non-selective autophagy and selective autophagy. However, the characters of these pathways are at the relatively early stages [121].The formation of autophagic vesicle carry on initiation, elongation and maturation step by step and subsequently fusion with lysosomes to form autolysosome which captures cellular contents and targets them for degradation. Provided that cells are absence of essential nutrients, autolysosome degrades membrane lipids and proteins for free macromolecules which can be recycled to generate energy and maintain protein synthesis. The morphological feature of autophagy-dependent cell death is autophagic vacuolization which is also commonly occurred in apoptotic or necrotic cell death, and currently no protein apart from the core autophagy proteins have been considered to be important for autophagy-dependent cell death [122]. In 2018, the NCCD defined it as a form of RCD that mechanistically depends on the autophagic machinery (or components thereof) [2].

Between the 1960s and 1980s, most scientists made much progress in understanding how cell produce proteins. Although De Duve and his colleagues firstly discovered the lysosome in 1950s, a few scientists were interested in protein degradation [123]. After a few years, in 1962, T.P. Ashford and K.R. Porter firstly discovered autophagy through the electron microscope that massive cytoplasmic components were destroyed in hepatic cell lysosomes [124]. Subsequently, in 1963, De Duve put forward the term ‘autophagy’ at the CIBA Foundation Symposium on Lysosomes. In 1967, De Duve and Deter found that after the injection of a large dose of glucagon, a robust inducer of autophagy in liver, a growing number of rat-liver lysosomes participated in this autophagy [125]. In 1973, Robert Bolender and Ewald Weibel presented the first evidence autophagy selectively sequestrated an organelle, the smooth endoplasmic reticulum [126]. Notably, autophagy is part of the lysosomal system as a degradative mechanism. In the early 1980s, Aaron Ciechanover, Avram Hershko and Irwin Rose discovered the ubiquitin-mediated protein which now consider to be a fundamental biological mechanism of protein degradation [127]. Provided that protein degradation broke down, such as the mutations of autophagy related genes, it will cause aberrations in pathogenesis of human diseases. Autophagy plays the important role in protein quality control by degrading accumulation of damaged and pathologic proteins in human diseases, especially neurodegenerative diseases.

There are multiple genes and proteins participate in autophagy progress that every protein is responsible for regulating different steps of autophagosome biogenesis. In 1990s, Yoshinori Ohsumi identified the key autophagy-related genes (ATG) using genetic screen for autophagy mutants in yeast [128, 129]. Although increasingly ATG genes have been reported, there are 15 genes well-known as core ATG genes contributed to the fundamental mechanism for the biogenesis of autophagy-related membranes [130]. In 1992, Yoshinori Ohsumi’s laboratory revealed the morphology of autophagy in yeast and first demonstrated that under yeast nutrient-deficient conditions, the vacuoles of yeast cells caused extensive autophagic degradation of cytosolic components [131]. With the increasing number of ATG protein discovered, understanding the function of ATG proteins in cell death will provide us more knowledge of autophagosome biogenesis. Whereas, the regulation of autophagy in human cells still remains largely unknown.

### The features and pathway of autophagy-dependent cell death

Autophagy-dependent cell death, a mechanism of cell death that is distinct from apoptosis or necrosis. Autophagy is a catabolic process of various cytoplasmic components, such as protein aggregates and organelles. Through autophagy, the release of nutrients is recycled in metabolic reactions. These components are marked as autophagy substrates and then phagocytoses by autophagosome, which can fuse and degrade with the lysosome. This depends on a large number of ATG genes, which are conserved from yeast to human [122, 132]. The novel formation of initiation complex in the process consists of the ULK1 complex (also known as the ATG1 complex in yeast) with ULK1, FIP200, ATG13 and ATG101, regulatory class III PI3 kinase complex with Beclin-1 (also known as ATG6) and ATG5-ATG12-ATG16 multimerization complex [133–135]. ATG9, a sole transmembrane protein in the autophagosome-forming progress, is recruited by ATG1-ATG13 complex and crucial for the initial lipidation of the phagophore membrane [136]. The complete autophagosome is marked by the release of LC3 II from the exterior surface of the membrane, which is then recycled. Thus, LC3 II is a prominent index used to monitor autophagic flux [137]. As a multistep process, autophagy can be inhibited or induced at different steps. For instance, MTOR inhibitors rapamycin or Torin1 are known as autophagy inducer. By contrast, Chloroquine (CQ) and its derivatives (such as 3-hydroxychloroquine) are utilized as autophagy inhibitor. The inhibition mechanism is to increase the lysosomal pH and ultimately suppresses the fusion between autophagosomes and lysosomes, resulting in preventing the maturation of autophagosomes into autolysosomes and inhibiting autophagy degradation [138].

**Fig. 3:**
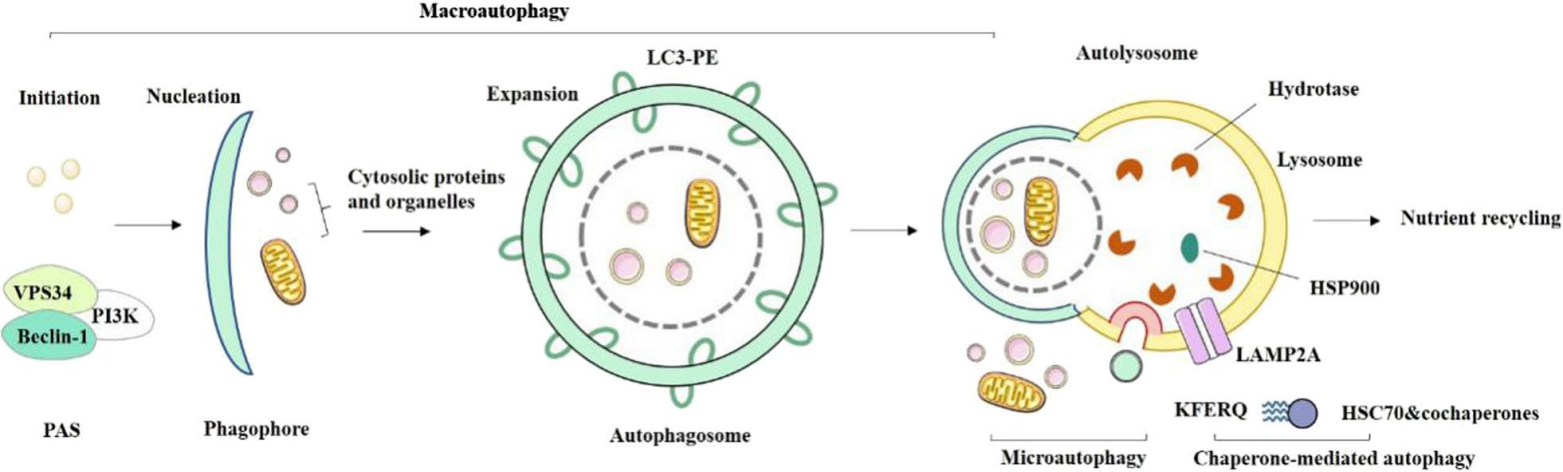
Schematic pathways of mammalian autophagy. In macroautophagy, the initiation of autophagy begins with the formation of the phagophore assembly site (PAS) and signals the activity of the vacuolar protein sorting 34 (VPS34) complex. Further nucleation requires a class III PI3K complex, which is composed of VPS34, PI3K and beclin-1. PE- conjugated LC3 (LC3-PE) is necessary for autophagic membrane expansion, recognition of autophagic substances, and fusion of autophagosomes with lysosomes. The resulting autophagosomes fuse with endocytosis and lysosomal compartments, ultimately leading to the formation of autolysosome. In microautophagy, the substrate is directly swallowed by the boundary of the lysosomal membrane. Then, the sequestration of cargo forms a lumenal vesicle by the protrusion and/or invagination of the vacuolar membrane. This vesicle is subsequently degraded by vacuolar hydrolases releasing simple decomposition products. In chaperone-mediated autophagy, the substrate with the pentapeptide motif KFERQ is selectively recognized by the heat shock cognate 70 kDa protein (HSC70) molecular chaperone and translocates to the lysosome in a LAMP2A-dependent manner. Proteins with exposed KFERQ or KFERQ-like motifs are recognized and bound by HSC70. The complex then localizes to the lysosomal membrane where the multimerization of LAMP2A allows the formation of aconitum to delivery the protein into the lysosomal lumen, a process facilitated by the lumenal chaperone HSP90

As shown in Fig. 3, macroautophagy, a catabolic process, degrades the cytoplasmic components, protein aggregates and organelles, and participate in the formation of autophagosomes with double membrane-bound vesicles for the phagocytosis of cytoplasmic proteins and organelles. Autophagosomes are transported to lysosomes where the sequestered cargo is degraded [139]. Microautophagy can be simply described as the invagination of the lysosomal or endosomal membrane, which cause directly engulf substrates and subsequent degradation by lysosomal proteases. CMA is distinct with macroautophagy and microautophagy since the cargo is not sequestered within a membrane delimited vesicle [140]. Instead, specific proteins containing a KFERQ-like pentapeptide motif are targeted by CMA which can bind the molecular chaperone heat shock cognate 70 kDa protein (HSC70). HSC70 increases substrate affinity and facilitates the translocation of these substrate proteins through lysosomal membranes into the lysosomal lumen via the lysosomal associated membrane protein 2A (LAMP2A) receptor [141]. In the present study, a simplified schematic pathways of mammalian autophagy definition were summarized (Fig. 3).

In recent years, selective autophagy induced by autophagy substrates has drawn more and more attention. Selective autophagy is mediated by autophagy cargo receptors that bind cargo marked with degradation signals, which most universal is ubiquitin in mammals, through their ubiquitin-binding domain (UBD). Autophagy cargo receptors serve as molecular bridges to capture ubiquitylated proteins targeted for degradation of cargos and complement of the ubiquitin–proteasome system (UPS). For instance, the autophagy cargo receptors p62/SQSTM1 and histone deacetylase 6 promote the autophagic selective removal of protein aggregates in a process termed as aggrephagy which is dependent on both the UBD and LC3-interacting region (LIR) [142]. Selective autophagy interacts with the autophagic substrate and the autophagosome via an LIR, then mobilizes specific metabolites in response to various cellular requirements. ATG5 is a gene product which is required for the formation of autophagosomes. Interestingly, some studies show that ATG5 may play a dual role in the modulation of autophagy and apoptosis. ATG5 interacts with FADD through its C terminal domain and promote apoptotic cell death. In addition, calpain-dependent cleavage of ATG5 removes the carboxyterminal domain of ATG5 which then generates a pro-apoptotic components that translocate to the mitochondria and induce the intrinsic apoptosis. Meanwhile, one study found that Beclin-1 cleaved by caspase-3 inhibits autophagy and promotes apoptosis [143–145]. Thus, it can be inferred that autophagy is mediated by negative modulation of apoptosis in some circumstances. By contrast, apoptotic signaling can be utilized to inhibit autophagy. Although the sophisticated mechanisms mediating the counter-modulation of apoptosis and autophagy has not been fully understood, the close link between autophagy and apoptosis is without no doubt.

### Autophagy-dependent cell death and neurodegenerative diseases

As an evolutionarily conserved degradation pathway, autophagy is related to human diseases and aging, especially neurodegenerative diseases [132, 146]. Selective autophagy targets damaged organelles, intracellular pathogens and protein aggregates to control the quality of the cytoplasm components by eliminating pathogenic proteins and organelles [147, 148]. Deficiencies in the autophagy-lysosomal pathway can contribute to the aggregation of abnormal protein, the generation of toxic substances and the accumulation of dysfunctional organelles [149]. Due to their extreme polarization, size and postmitotic properties, neurons may be particularly vulnerable to accumulation of aggregated or damaged cytoplasmic compounds, and rely on autophagy for cell survival in physical [150]. Genetic studies have showed that highly tight connections between autophagy and neurodegenerative diseases including AD, PD, ALS and HD [151].

Evidence has suggested that huntingtin, the specific protein in HD, contribute to macroautophagy [152]. In the HD models, anomalous mitochondria cannot be engulfed by autophagosomes. The main function of the mHTT is interacting with autophagy receptors and blocking them from binding to damaged mitochondria [153].The expansion of the polyglutamine (polyQ) tract in the N-terminus of the huntingtin (HTT) protein gives rise to protein aggregation [154]. Selective autophagy contributes to the removal of the mHTT, but mHTT interrupts the dynamics of autophagy via the formation of autophagic vacuoles which caused the accumulation of mHTT and subsequent neurotoxicity [153, 155, 156]. HTT is also considered as CMA substrate, and phosphorylation-regulated CMA can enhance the degradation of normal HTT. Overexpression of HSC70 or LAMP2A increased HTT degradation, while knockout of these genes in the cell model decreased HTT degradation [157]. In vitro studies showed that TFEB overexpression enhanced ALP and reduced HTT protein aggregation in Huntington protein expressing cells by polyglutamine expansion. TFEB was also identified as a downstream mediator and transcriptional target of PGC-1α, which was shown to improve neurological function when overexpressed in HD mouse model [158, 159]. In addition, animal models of ALP targeted drugs (such as CCI-779, rimantadine and trehalose) in the treatment of HD have been proved to have therapeutic effects [155, 160, 161]. Recently, emerging studies have indicated that that autophagy is related with ALS that autophagy induced by rapamycin increases motor neuron degeneration in the mouse model of familial ALS [122, 162]. Enlarged autophagosomes containing p62 positive aggregates have been observed in ALS mouse models and ALS patients [163, 164]. Motor neuron specific ATG7 knockout mice bearing SOD1 pathogenic mutations have accelerated neuromuscular junction disruption and tremors, which are features of ALS [164]. Silencing of TDP-43 or ALS-associated mutations increases the transcription of BCL-2 and abnormal ATG4B protein, resulting in autophagy defects. Autophagy activation reduces TDP-43 aggregation and improves the survival rate of human motor neurons bearing TDP-43 mutation [165–168]. Moreover, mutations in the gene encoding SOD1 lead to the occurrence of familial ALS, causing misfolding, aggregation and accumulation of proteins and the progressive loss of motor neurons [169].

AD is the most common neurodegenerative disease, which is characterized by Aβ. Extracellular amyloid plaques and intracellular neurofibrillary tangles (NFT) composed of hyperphosphorylated tau protein. AD may have a direct genetic origin, such as mutations in amyloid precursor protein (APP) and presenilins (PS) 1 and 2. Loss of function or AD associated mutations in PS1 have been shown to cause V-ATPase V0a1 subunit maturation and failure of V-ATPase complex assembly, which is required for lysosomal acidification and protease activity [170, 171]. Postmortem studies of human samples showed that there was the accumulation of autophagosomes, multivesicles and autolysosomes in dystrophic neurites [172]. TFEB mediated beneficial effects have been confirmed in a variety of AD and tau pathological mouse models. Enhancing ALP through the expression of exogenous TFEB in the brain can significantly reduce tau pathology, neurodegeneration and behavioral defects in rTg4510 mouse model [173]. The expression of TFEB in astrocytes promotes the reduction of Aβ plaque lesions in the APP/PS1 mouse model through uptake and lysosomal degradation of Aβ [174]. PD as an aging-related neurodegenerative diseases, accumulating evidence suggests that autophagy-dependent cell death is relevant to PD pathology [175, 176]. Autophagy is relevant to the regulation of the inflammatory reaction, PD is characterized by not only dopaminergic neuron degeneration, but also microglia-mediated neuroinflammation. Selective autophagy can contribute to microglia activation which can regulate IL-1β and IL-18 gener by NLRP3 degradation, suggesting that the details of impaired autophagy could give rise to neuroinflammation in PD [177, 178]. Several studies have revealed that defective mitophagy is closely linked to PD. Dysfunction of normal autophagy/mitophagy may can cause mitochondrial malfunction and thereby promote neuron death. PINK1 and PRKN show loss-of-function mutations in autosomal recessive juvenile parkinsonism [179]. PINK1 (a mitochondrial protein kinase) and Parkin (an ubiquitin E3 ligase) have been genetically related to mitophagy that removes damaged mitochondria and blocks progressive mitochondrial dysfunction [139, 176]. The beneficial effect of autophagy in the nervous system is mainly related to maintaining a normal balance between cellular protein formation and degradation. Recently, a study has been reported that modulating TRADD, as a novel therapeutic target in vitro and vivo block both apoptosis and inflammation, simultaneously activate autophagy in order to maintain cellular homeostasis through the removal of pathologic protein aggregations [180].

Although hereditary neurodegenerative diseases are caused by various gene mutations, the accumulation of protein aggregates is their common feature. A large number of studies have shown that several genes related to these diseases are involved in the autophagy-lysosome pathway, and intracellular protein aggregates can destroy several steps of autophagy. Therefore, we speculate that up-regulation of autophagy can improve neurodegenerative diseases. Autophagy activation reduced the accumulation of inclusion bodies and further alleviates the neurodegenerative phenotype. However, the use of autophagy inducers to interfere with neurodegenerative diseases is still in its infancy. Most of the currently used pharmacological autophagy regulation strategies are based on the overall induction of the entire autophagy process. In addition, excessive autophagy activation can lead to toxic effects. For this reason, it is necessary to thoroughly understand the role of autophagy in various neurodegenerative diseases.

## Necroptosis

### The definition and discovery of necroptosis

Previously, cell death mechanisms were inaccurately divided into two types: PCD like apoptosis and necrotic cell death. The mechanisms of PCD require energy, while the mechanisms necrotic of cell death do not. Importantly, the typical character necrotic cell death gives rise to a strong immune response, whereas PCD does not [181]. Necroptosis, a programmed form of necrosis with the morphological features similar to necrosis, is a pathway which is necessary for cell survival, inflammation and diseases [17, 182]. Necroptosis is regulated by the RIPK1 and receptor-interacting kinases 3 (RIPK3) and their substrate is mixed-lineage kinase domain-like protein (MLKL), facilitating its oligomerization and activation [183]. Necroptotic cells take place rapid membrane permeabilization through the executioner protein MLKL and subsequently mediate the release of intracellular contents [14, 184]. Caspase-8 negatively regulate this type of cell death [185]. Necrostatin-1 (Nec-1) is a small molecular inhibitor of necroptosis, which blocks the activation of RIPK1 [186]. In 1988, it was reported that TNF can trigger both apoptosis and a unknown form of PCD with the typical morphology of necrosis [187]. In 1996, the finding reported the porcine kidney cells with the infection of cowpox virus induced a necrotic cell death only provided that cells expressed the viral caspase inhibitor cytokine response modifier A. Therefore, it can be inferred that necroptosis is executed independently of caspases [188]. In the late 1990s, studies strengthened the hypothesis that deficiency of caspase signaling could trigger programmed form of necrosis. The study revealed that inhibition of caspases promote the sensitivity of L929 cells to necroptosis mediated by TNF [189]. Another study demonstrated that Fas receptor can trigger two different pathways of cell death, one directly and rapidly leading to apoptosis, and the other causing the cells to necrosis and the production of oxygen radical, when apoptosis is hindered by caspase inhibitors [190]. In 2005, the programmable and regulated new necrotic cell death was named ‘necroptosis and these findings are the cornerstone for further study in the new area [186]. In 2008, the study identified that RIPK1 is the key upstream kinase participating in the activation of necroptosis pathway [191]. Subsequently, in 2009, the study discovered that after the induction of necroptosis, RIPK1 recruited RIPK3 to form a necrosis-inducing complex referred to as necrosome [192]. In 2012, the study demonstrated that knocking down MLKL expression protect cell against necroptosis, suggesting that MLKL is a key molecule of signaling downstream of RIPK3 in necroptosis [193]. In 2018, NCCD defined necroptosis as a type of RCD triggered by perturbations of extracellular or intracellular homeostasis that critically depends on MLKL, RIPK3, and (at least in some settings) on the kinase activity of RIPK1 [2]. Notably, necroptosis, pyroptosis and ferroptosis are regarded as the three novel mechanisms of immunogenic cell death which are considered to be a defense against infection and are highly related with antitumor immunity [184, 194].

### The features and pathway of Necroptosis

Necroptosis is a novel pathway of programed necrosis which is executed under specific stimuli and involves activation of cell signaling pathways. Morphologically, necroptosis has the hallmarks like necrosis, such as swelling organelles and cells, rupture of the plasma membrane and release of the intracellular components and without the pyknosis seen upon chromatin condensation in apoptosis [2]. Main factors include RIPK1, RIPK3, and MLKL involved in necroptosis-related signal transduction [191, 195]. TNF-dependent signaling induced the formation of necroptosis-specific protein complex has been studied in depth. Necroptosis can be triggered by death receptors, interferons, toll like receptors, intracellular RNA and DNA sensors, and potentially other signal molecules. The identification of necrostatins targeting for RIPK1 to inhibit necroptosis provides evidence that TNF-induced necrosis is a kinase-regulated process.

**Fig. 4:**
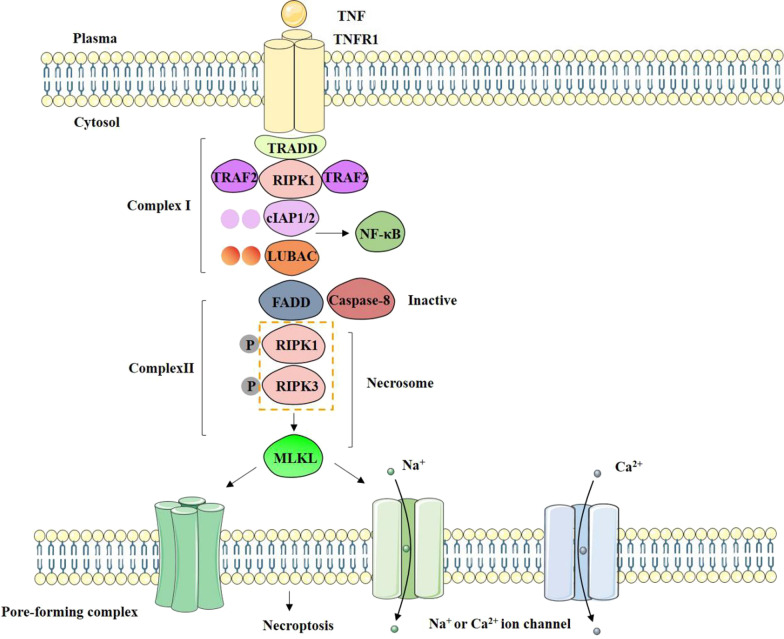
Schematic presentation of necroptosis pathway induced by tumor necrosis factor (TNF). The binding of TNF to its cognate receptor TNFR1 triggers the assembly of complex I, which includes TNFR1, TNFR1-associated death domain (TRADD), receptor-interacting serine/threonine protein kinase 1 (RIPK1), TNFR-associated factor 2 ( TRAF2), cellular inhibitor of apoptosis protein 1/2 (cIAP1/2), and linear ubiquitin chain assembly complex (LUBAC). Complex I provides a platform for a series of ubiquitination and deubiquitination reactions. This ubiquitination is related to nuclear factor-κB (NF-κB) or the decision between survival signals and cell death signals. Subsequently, the ubiquitination of RIPK1 by cIAP1 and cIAP2 stabilized complex I and made the further recruitment of additional factors. The cytoplasmic death-inducing signal complex composed of RIPK1/3, MLKL, caspase-8 and FAS- associated death domain protein (FADD) is called complex II. When RIPK3 and MLKL levels are sufficiently high and caspase-8 activity is inhibited, complex II may evolve to form necrosome. Upon receipt of a necroptosis-inducing stimulus, RIPK1 phosphorylates and activates RIPK3, which in turn phosphorylates and activates MLKL, forming a complex called necrosome. Then, MLKL is recruited and phosphorylated by RIPK3 to form active oligomers. The executor of necroptosis is MLKL, there are two non-exclusive models are proposed for the mechanism of MLKL. One could act directly as a direct pore-forming complex that is recruited through binding of the amino-terminus, another could act indirectly by serving as a platform that deregulates Ca^2+^ or Na^+^ ion channels

Necroptosis is activated under apoptosis-deficient conditions [186]. The response of cell to TNF is multiple and TNF can trigger either apoptosis or necroptosis. For the most part, necroptosis contributes to the activation of nuclear factor-κB (NF-κB) and mitogen-activated protein kinase (MAPK) signaling. As shown in Fig. 4, when TNF binds to its receptor TNFR1, a receptor-associated ‘Complex I’ involved with RIPK1, TNFR1, TRADD, TNFR-associated factor 2 (TRAF2), the linear ubiquitin chain assembly complex, cellular inhibitor of apoptosis protein 1 (cIAP1) and cIAP2. Complex I provide the platform for a range of ubiquitylation and deubiquitylation reactions to manipulate the switching among NF-κB signaling with cell survival signals and cell-death-inducing signals [186, 191]. RIPK3 is the downstream mediator of RIPK1 in necroptosis process [192]. The necrosome is a complex consisting of RIPK1 and RIPK3 to participate in the activation of necroptosis. The important study identified the MLKL is the downstream of RIPK3 activation by using a chemical screen [193]. The involvement of MLKL with the plasma membrane rupture is essential for cell death and MLKL is the effector of necroptosis. Activated RIPK3 phosphorylates and recruits MLKL to assemble a protein complex at the plasma membrane. First, these oligomers can directly promote pore-forming, contributing to plasma membrane destabilization. Second, they can indirectly serve as a platform to deregulate Ca^2+^ or Na^+^ ion channels. There exists six human death receptors (DRs) in the TNF superfamily, including TNFR1, FAS (also known as CD95 or APO-1), DR3 (also known as TRAMP or APO-3), DR4 (also known as TRAILR1), DR5 (also known as TRAILR2, TRICK or KILLER), and DR6 [195, 196]. In the present study, a simplified schematic presentation of necroptosis pathway induced by TNF definition were summarized (Fig. 4).

### Necroptosis and neurodegenerative diseases

Necroptosis can be triggered under pathological conditions such as inflammatory, infectious diseases and neurodegenerative diseases. However, it has become increasingly apparent that necroptosis is often not activated alone. The deleterious effects of TNF-α may cause the activation of both necroptosis and RIPK1-dependent apoptosis in the same time, which the contributions of each pathway and their interactions may change in different pathological conditions [197]. Transgenic models and pharmacologic inhibition have demonstrate that RIPK1, RIPK3 and MLKL are involved in many neurodegenerative diseases [198].

Evidence of necroptosis was found in post-mortem examination of human AD patients, and MLKL expression was abundant compared with healthy controls. Necroptosis was exacerbated cognitive deficits in AD APP/PS1 mouse models, treatment with RIPK1 inhibitor Nec-1 reduced neuronal death and insoluble Aβ in cortex and hippocampus plaque and hyperphosphorylated tau formation and ameliorated cognitive impairment [199, 200]. It is worth noting that, in addition to inducing necroptosis, RIPK1 (and RIPK3) are also involved in the activation of caspase-8-mediated apoptosis and the production of cytokines and chemokines [201]. It is not clear which process in APP/PS1 mice inhibited by Nec-1 reduces pathology. A study has shown that RIPK1 can promote the formation of microglia subtypes and Aβ plaques in AD patients to trigger inflammation and cause disease. In AD mouse models, drug inhibition or gene ablation of RIPK1 can reduce amyloid load, inflammatory cytokine levels and memory deficits. Therefore, RIPK1 is considered to be a promising target for AD therapeutic intervention [202].

One of the main signs of PD is the degeneration of dopaminergic neurons in the substantia nigra, and its pathogenic mechanism is thought to be the activation of programmed neuronal death. Necroptotic activation and miR-425 deficiency in the substantia nigra were observed in the brains of MPTP-treated mice and PD patients. Gene knockdown of miR-425 exacerbates MPTP-induced motor deficits and dopaminergic neurodegeneration through upregulation of early necrosis genes. Intracerebral miR-425 mimics (AgomiR-425) attenuated necroptotic activation and dopaminergic neuron loss, and improved motor behavior [203]. These findings identify miR-425 as a potential treatment for PD. In the preclinical model of PD, gene ablation of MLKL or RIPK3 or pharmacological inhibition of RIPK1 reduced the degeneration of dopaminergic neurons, improved motor ability and played a neuroprotective role. This is a drug pathway that targets the loss of dopaminergic neurons. Phosphorylated MLKL was found in post-mortem brain biopsies of human PD patients [204]. In the tissue culture model of PD, treatment withRIPK1 inhibitor can protect iPSC-derived neural cells from death and reduce oxidative stress in PD patients with optic atrophy type 1 (OPA1) gene mutation [205].

The role of necroptosis in the pathogenesis of HD is rarely reported. An early study reported that in the R6/2 transgenic HD mouse model, e*xon1* of the mutant human HTT gene was expressed and driven by the human huntingtin promoter [206].Treatment with Nec-1 can ameliorated symptoms and delayed disease progression in mice, and determine the role of RIPK1 in disease progression [207]. The results suggest that necroptosis may play a role in the pathogenesis of the disease, and RIPK1, the inducer of necrotic ptosis, may be a promising drug target for HD. A recent study showed, OE-MSCs inhibited apoptosis and necroptosis through the trophic-rich environment have a potency in dwindling the symptom associated with HD [208].

MS is a common neurodegenerative disease. The core pathophysiologic characteristics of MS are the loss of oligodendrocytes and demyelination. There are increasing evidences that RIPK1 mediates the harmful process of chronic neurodegeneration. A key similarity between acute injury and chronic neurodegeneration is the presence of neuroinflammation. TNF-α is a pro-inflammatory cytokine related to MS that can activate necroptosis, which is a necrotic cell death pathway regulated by RIPK1 and RIPK3 in the absence of caspase-8 [209, 210]. Nec-1s inhibition of RIPK1 ameliorated disease pathology, improved animal behavior, and reduced the increase in cytokines and immune cell recruitment induced by experimental allergic encephalomyelitis (EAE). RIPK1 is highly expressed in macrophages and microglia of EAE lesions. Nec-1s can inhibit the innate immune response in these cells, and blocking the activity of RIPK1 can regulate the inflammation and cell death of microglia [211, 212]. Necroptosis mediates oligodendrocyte degeneration induced by TNF-α and targeting RIPK1 protects against oligodendrocyte cell death in both animal models of MS and culture [213]. Therefore, RIPK1 inhibition may provide a potential therapeutic strategy for MS.

ALS is a deadly neurodegenerative disease with the features of progressive loss of upper and lower motor neurons. SOD1 gene plays an important role in the ALS pathology. Recent studies have showed the activation of RIPK1, RIPK3 and MLKL in the spinal cords of SOD1^G93A^ mutant mice and in human ALS models, suggesting necroptosis may be implicated in the pathology of ALS [52, 214]. Furthermore, this hypothesis is verified by using Nec-1 and RIPK3 knockout, motor dysfunction onset was delayed and axonal myelination defects were blocked in SOD1^G93A^ mutant mice [52, 214]. Thus, we cannot exclude the implication of necroptosis to the pathology of ALS and motor dysfunction in the SOD1^G93A^ mutant mice. However, recent study presents the questioned view that knockdown of MLKL in SOD1^G93A^ mutant mice does not influence either motor neuron degeneration and neuroinflammation, or the development and progression of ALS [214]. Furthermore, mutations in the optineurin (OPTN) gene have also been associated with both familial and sporadic ALS. The study showed that OPTN deficiency in the spinal cord of mice caused RIPK1-dependent inflammation and axonal degeneration. Therefore, the relevance of necroptosis and ALS still remains unclear and plays an important role in treating diseases.

In theory, necroptosis can be inhibited on multiple sites, such as targeting for RIPK1, RIPK3 or MLKL. However, most experimental studies focus on cell death forms as independent one. Their molecular mechanism and signal pathway are highly connected, but also might be complementary and mutual restrictions in their effects of human cells. It should be considered that MLKL deficiency offered less benefit in some animal models, suggesting that MLKL might not be an excellent target for the treatment of neurodegenerative diseases [215, 216]. The inhibition of RIPK3 may result in apoptosis which limits the potential of RIPK3 inhibitors for therapeutic benefit [217, 218]. Above all, these findings suggest that targeting multiple cell death key sites would be more effective than single therapy approach and targeting RIPK1 may provide an promising therapeutic strategy for the treatment of neurodegenerative diseases [215].

## Ferroptosis

### The definition and discovery of ferroptosis

Ferroptosis is a unique iron-dependent form PCD with the hallmark of accumulation of intracellular ROS. In 2012, Dixon, etc. found that erastin and oncogenic RAS selective small molecule lethal 3 (RSL3) reagents can specifically trigger ferroptosis [219]. When cells are treated with ferroptosis inducing agents, mitochondria shrink and mitochondrial cristae disappear. It has also a series of changes in the biochemical characteristics, such as cell membrane lipid peroxide accumulation and reduced glutathione (GSH) depletion. Ferroptosis can be prevented by enzymatic reactions of two major antioxidant systems, including glutathione peroxidase 4 (GPX4) catalyzing the reduction of lipid peroxides in a GSH dependent reaction, and the recently discovered ferroptosis inhibitory protein ferroptosis suppressor protein (FSP1) catalyzing the regeneration of ubiquinone [220]. Interestingly, some study found that ferroptosis is a type of autophagy-dependent cell death [221]. The harmful effects of ferroptosis can be inhibited by iron chelators such as deferoxamine (DFO), and lipid peroxidation inhibitors such as vitamin E, ferrostatin-1 (Fer-1), and liproxstatin-1 (Lip-1) [219, 222]. However, apoptosis, necrosis and other PCD inhibitors cannot inhibit cell death induced by erastin and RSL3. In 2003, erastin was discovered as the first ferroptosis inducer by using high-throughput screening of small-molecule libraries. Erastin-induced cell death performs normally under non-apoptotic, RIPK1/RIPK3 silence and pharmacological inhibition of RIPK1 [223]. Subsequently, RSL3 was proved as ferroptosis inhibitor [224]. Therefore, the new form of cell death induced by erastin and RSL3 is distinct from other reported RCD [225]. In 2014, Yang et al. reported that GPX4 plays a key role in the prevention of ferroptosis by reducing phospholipid hydroperoxide, thereby inhibiting lipoxygenase mediated lipid peroxidation [226]. In 2017, it was shown that acyl CoA synthetase long chain family member 4 (ACSl4) is a biomarker and key initiator of ferroptosis, which is required for the production of polyunsaturated fatty acids (PUFA), and PUFA is required for the execution of ferroptosis [227]. Further research in 2018 described the requirement for GPX4 to utilize selenium to suppress ferroptosis [228]. In 2018, the NCCD define ferroptosis as a form of RCD initiated by oxidative perturbations of the intracellular microenvironment that is under constitutive control by GPX4 and can be inhibited by iron chelators and lipophilic antioxidants [2]. Recently, it was discovered that the coenzyme Q10 oxidoreductase FSP1 can inhibit ferroptosis in a glutathione-independent manner, thus establishing a new ferroptosis inhibitory pathway [220].

### The features and pathway of ferroptosis

Ferroptosis is a consequence of accumulated iron and lipid peroxidation. This morphological process is cell volume shrinkage, membrane damage, increased mitochondrial membrane density, dysmorphic small mitochondria with decreased crista without typical apoptotic and necrotic features, such as the release of cytochrome C from mitochondria, caspase activation, and chromatin fragmentation [225, 229]. The key biological features of ferroptosis include depletion of GSH and iron-mediated lipid peroxidation [230]. The classical pathway triggers ferroptosis by inhibiting one of the two major antioxidant systems. One is the antiporter, System X_c_^−^, consisting of disulfide-linked heterodimers SLC7A11 and SLC3A2, uptakes adequate cystine (the extracellular oxidized form of cysteine), in transport for intracellular glutamate. Cystine is necessary for GSH synthesis. GSH, a tripeptide anti-oxidant, serves as an essential cofactor of GPX4 to detoxify lipid hydroperoxides [231]. Another antioxidant system is GPX4, a phospholipid hydroperoxidase, which is the key regulator of ferroptosis. GPX4 can directly reduce phospholipid hydroperoxide production by catalyzing the GSH-dependent reduction lipid peroxides. Inactivated GPX4 through direct or indirectly targeting pathways can trigger ferroptosis. While inhibition or depletion of GPX4 directly, depletion of intracellular GSH indirectly inactivates GPX4 [226, 231]. Non-canonical ferroptosis refers to ferroptosis initiated by increasing the intracellular labile iron pool due to overactivation of heme oxygenase-1 [232]. When inhibiting the synthesis of GSH or the GSH-dependent antioxidant enzyme GPX4 in vivo and vitro, ferroptosis is triggered. In various cell types, including neurons, GPX4 as an antioxidant enzyme plays an important role in inhibiting excessive lipid peroxidation and GPX4 inhibitor RSL3 can trigger ferroptosis [2].

**Fig. 5:**
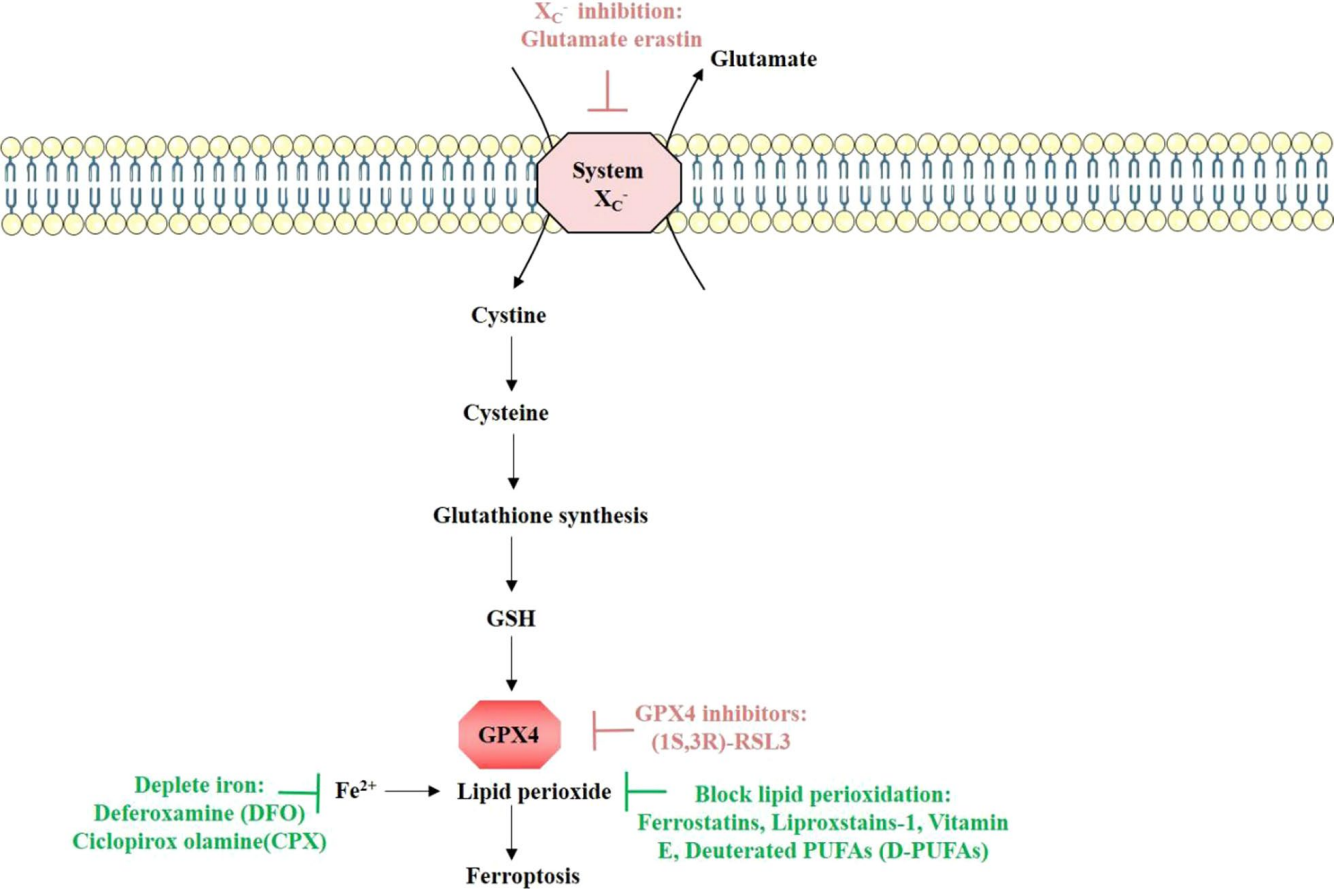
Ferroptosis is a novel form of cell death dependent on iron and lipid peroxidation. Inhibition of system Xc- and glutathione peroxidase 4 (GPX4) causes ferroptosis. Glutamate receptors activate or inhibit the Xc- system, the glutamate/cystine antiporter, which can cause glutamate-induced toxicity. The uptake of extracellular cysteine in the form of cystine is a key step in the synthesis of GSH, and GSH generation and maintenance is significant for preventing cells from the damaged oxidative stress responses. The depletion of glutathione or GSH levels affect the function of GPX4, which is a member of the GSH peroxidases. GPX4 inactivation gives rise to the accumulation of lipid peroxides and ferroptosis. Blocking up lipid peroxidation and iron chelation are the inhibitor of ferroptosis. Small-molecule inducers of ferroptosis are colored red, small-molecule inhibitors of ferroptosis are colored green

Glutamate and glutamine are important modulating factors for ferroptosis. Glutamine is normally kept at high concentration in human tissues and serum. Glutaminolysis can provide energy for the Krebs cycle and hinder some synthesis reactions (such as lipid synthesis). Glutaminolysis is essential for ferroptosis triggered by deprivation of cysteine [233, 234]. As shown in Fig. 5, by the cystine/glutamate antiporter system (system X_c_^−^), glutamate in the cell is replaced with cystine according to the proportion of 1:1. High extracellular glutamate concentration can inhibit the function of the system X_c_^−^ and lead to cell death because excessive glutamate accumulation causes intracellular cysteine imbalance. In the case of glutamine deficiency or blocking glutamine synthesis, intracellular cystine deficiency and blocking of cystine input cannot induce ROS accumulation, lipid peroxidation and ferroptosis [233]. Lipid metabolism is also strongly implicated in ferroptosis. Peroxidation of PUFAs are sensitive to lipid peroxidation in the occurrence of ferroptosis. Supplementing cells with PUFAs to prevent this peroxidation suppresses ferroptosis. The content and location of PUFAs determine the extent to lipid peroxidation, and thereby the severity of ferroptosis [235]. Iron is one of the essential elements for the accumulation of lipid peroxide and the process of ferroptosis [236]. Iron can produce excessive ROS through Fenton reaction to promote lipid peroxidation in ferroptosis, causing DNA and lipid damage. Iron also can promote the activity of non-heme iron-containing enzymes, for example, lipoxygenases promoting the lipid peroxidation [237]. Therefore, proteins associated with the input, excretion, storage, and circulation of iron can affect the occurrence of ferroptosis. These proteins include iron responsive element binding protein 2, transferrin, transferrin receptor, nuclear receptor coactivator 4 (NCOA4) and divalent metal transporter 1 [225, 233, 238]. Abnormal or dysfunctional expression of these proteins give rise to increased concentration of iron ions and metabolic disorders. Excessive iron accumulation in tissues leads to accumulation of ROS and lipid peroxide, causing ferroptosis. This process is regulated by IREB2, and the silence of IREB2 can inhibit the occurrence of ferroptosis [235]. Indeed, iron chelators block the onset of ferroptosis in vitro and in vivo, such as DFO, Deferiprone, Deferasirox [225, 239]. Induction of ferroptosis usually increase cellular labile iron [240]. In addition, supplying exogenous sources of iron enhance the sensitivity of cells to ferroptosis inducers [219]. Despite intense studies on iron involved in ferroptosis, the role of iron in ferroptosis still remains unclear. In the present study, a simplified ferroptosis, an iron-and lipid peroxidation dependent form of cell death definition was summarized (Fig. 5).

**Fig. 6:**
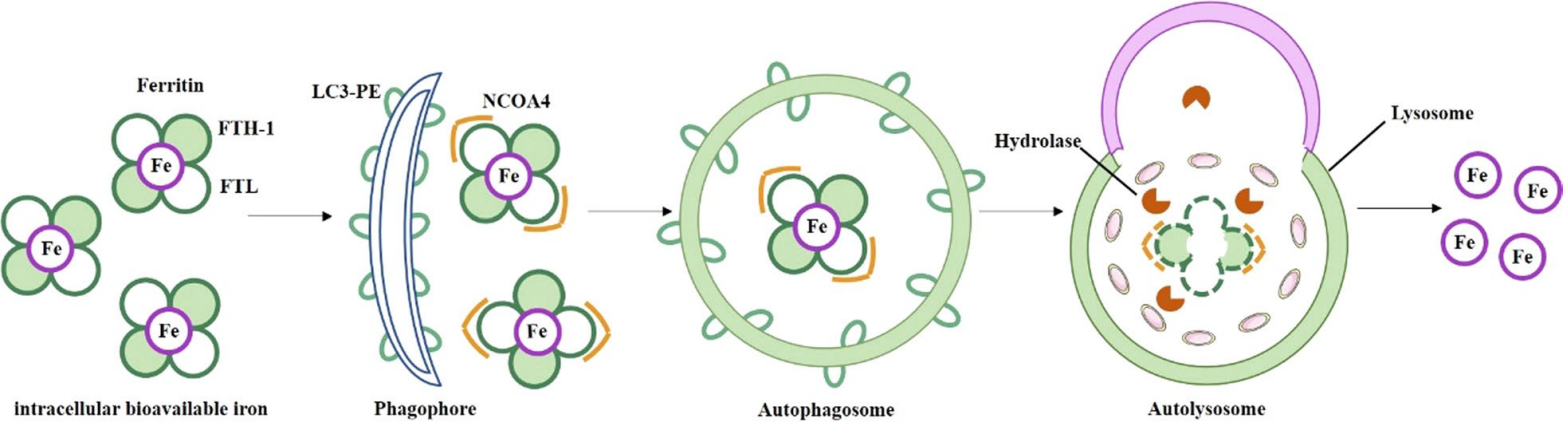
Signaling pathway mediated by ferritinophagy. In condition of starvation or iron depletion in the cell, ferritinophagy identifies nuclear receptor co-activator 4 (NCOA4) as a specific autophagy cargo receptor, binds ferritin and targets it for lysosomal degradation. Ferritin is a major intracellular iron storage protein complex, which includes ferritin light chain (FTL) and ferritin heavy chain 1 (FTH1). NCOA4 is a cargo receptor that recruits ferritin to autophagosome by binding FTH1 and sequesters ferritin complexes into autophagosomes by binding to microtubule-associated protein 1 light chain 3-phosphatidylethanolamine (LC3-PE) with developing double-membrane of autophagosome. As autophagosome fully matured and fusion with the lysosome which releases hydrolase, both NCOA4 and ferritin are degraded in autolysosome, consequently releasing bioavailable iron

Autophagy can affect ferroptosis by regulating affecting iron metabolism. Ferroptosis is regarded as an autophagic cell death process and the selective autophagic turnover of ferritin is termed as ferrotinophagy which is involved in ferroptosis. Biochemical studies show that NCOA4 is a key mediated factor of ferritinophagy as an autophagy cargo receptor which binds and targets ferritin for lysosomal degradation [238]. As shown in Fig. 6, NCOA4-mediated ferritinophagy promotes ferroptosis by degradation of ferritin and controlling cellular iron homeostasis. The induction of ferroptosis contributes to autophagy activation and consequently degradation of ferritin and NCOA4. Inhibition of autophagy or knockdown of NCOA4 decreases the accumulation of cellular labile iron and ROS, eventually suppresses ferroptosis [240]. NCOA4 combines with ferritin heavy chain 1 (FTH1), colocalizes with cellular ferritin, and sequesters ferritin and iron complexes into autophagosomes by binding microtubule-associated protein 1 light chain 3-phosphatidylethanolamine (LC3-PE) on the unmature autophagosome membrane. When it comes to autophagosome formation and fusion with the lysosome, both NCOA4 and ferritin are degraded, consequently releasing bioavailable iron [241]. Despite NCOA4 lacks canonical LIR motif which is found in other autophagy cargo receptors, NCOA4 depletion inhibits the autophagy-dependent and lysosomal-mediated degradation of ferritin. Moreover, under circumstance of starvation or iron depletion, the deprivation of NCOA4 reduces the level of bioavailable intracellular iron and causes the accumulation of iron in splenic macrophages in vivo [242]. However, the interplay between autophagy and ferroptosis at the genetic level is still unclear. In the present study, a simplified signaling pathway mediated by ferritinophagy definition were summarized (Fig. 6).

### Ferroptosis and neurodegenerative diseases

Evidence has been found that ferroptosis plays a significant role in the occurrence and development of neurodegenerative diseases. Lipid peroxidation and iron homeostasis imbalance and accumulation are the two basic conditions of ferroptosis. Recently, ferroptosis leads to the loss of neurons in AD, which has attracted more and more attention [243, 244]. The imbalance of brain iron and the decrease of endogenous antioxidant system including GPX are closely related to the pathology of AD, accompanied by high Aβ The cortical iron content of mild cognitive impairment patients with plaque load is higher, and the brain iron level is positively correlated with the progress of AD and the decline of cognitive ability [245–247]. GPX4BIKO mice (a mouse model of conditional deletion of GPX4 in forebrain neurons) showed obvious defects in spatial learning and memory function and hippocampal neurodegeneration. These results are related to ferroptosis markers, such as lipid peroxidation, ERK activation and increased neuroinflammation. In addition, GPX4BIKO mice fed a vitamin E deficient diet accelerated the incidence of hippocampal neurodegeneration and behavioral dysfunction. When treatment with ferroptosis inhibitor Liproxstatin-1 improved neurodegeneration in these mice [248]. In an in vitro model, iron increased neuronal cell death in the presence of reduced GSH levels by reducing the activity of glutamate cysteine ligase [249].

Recent studies have reported that the midbrain of PD patients is characterized by high iron, low reduced GSH and lipid peroxidation, suggesting that the pathogenesis of PD is closely related to ferroptosis [250]. Jenner et al. compared the brain tissues of PD patients and normal people, and found that the GSH level and the reduced/oxidized glutathione (GSH/GSSG) ratio in the substantia nigra of PD patients was reduced, while other parts were normal [251]. Glutamate neurotoxicity can be inhibited by iron chelators and Fer-1, suggesting a possible target for inhibiting ferroptosis [221]. Targeting fer-1 to inhibit ferroptosis can improve motor behaviors in MPTP-treated mice, a well-established animal model of PD, and prevent dopaminergic neuron loss [252]. In addition, the existing studies have shown that lipid peroxidation can directly promote neurons in the substantia nigra to degenerative changes [253]. Another study suggests that Glenn ketone drugs can target for ACSL4 to block ferroptosis-sensitive polyunsaturated phospholipid synthesis. GPX4-ACSL4 double-knockout cells showed remarkable resistance to ferroptosis, suggesting that ACSL4 contributes to ferroptosis sensitivity [227]. Thus, it can be inferred that the key link between ferroptosis and PD is redox dysfunctions and lipid peroxide accumulation in patients’ brain, regulating lipid metabolism pathway and reducing lipid peroxide accumulation may hold promise as potential targets for PD treatment.

HD is an autosomal dominant neurodegenerative disease, which is characterized by highly selective and severe damage to the striatum, leading to choreographic movement, dystonia and progressive dementia. MHTT may cause oxidative stress and neurotoxicity to striatal neurons, and eventually lead to neuronal dysfunction and neuronal cell death [254, 255]. However, the pathological mechanism of HD is complex and has not been fully clarified. Some features of ferroptosis, such as iron accumulation, lipid oxidation, oxidative stress, and GSH redox cycle disorders, have been observed in HD patients and experimental animal models [256–259]. Magnetic resonance imaging shows iron accumulation in the brain of HD patients [260]. A study on HD mouse model showed that toxic iron accumulated in neurons compared with wild model, suggesting that iron accumulation may contribute to the process of neurodegeneration [261]. Another study on GPX4 ablation mice showed that GPX4 ablation induced degeneration of spinal cord motor neurons showed the characteristics of ferroptosis, including no caspase-3 activation, no TUNEL staining, ERK activation and elevated spondylitis. Vitamin E supplementation, another ferroptosis inhibitor, can delay the occurrence of paralysis and death induced by GPX4 ablation [262]. Under normal conditions, GSH regulates the activity of GPX4, inhibits ferroptosis and eliminates excessive lipid peroxides. However, the increase of ROS level and lipid peroxide resulted in the consumption of GSH and decreased GPX4 [263]. The study of ferroptosis and its relationship with HD will undoubtedly help to further understand the pathogenesis of the disease and find more effective therapeutic targets, although there are still many unsolved problems in this field.

Since ferroptosis was first discovered in 2012, there are relatively few studies on ALS and MS. MS is a chronic demyelinating disease of the CNS and cuprizone (CZ) is a copper chelator that induces demyelination. Administration of CZ to mice in the diet resulted in the expression of oligodendrocyte ferroptosis markers in the corpus callosum. The administration of ferroptosis small molecule inhibitors can prevent CZ induced oligodendrocyte loss and demyelination. This work has a broader impact on diseases such as MS and CNS injury [264]. In MS gray matter and spinal cord of MOG35-55 peptide-induced EAE, the mRNA expression of all three GPX4 subtypes (cytoplasm, mitochondria and nucleus) decreased, and the number of GPX4 protein decreased. At the same time, there are two other ferroptosis negative regulators in EAE, which are important to maintain the physiological level of glutathione (γ-Glutamylcysteine ligase and cysteine/glutamate transporter) were also reduced [265]. The degeneration and death of ALS motoneurons are related to the increase of lipid peroxidation, which is the driving factor of ferroptosis. Compared with the control SOD1^G93A^ mice, SOD1^G93A^ GPX4 mice had extended lifespans, delayed onset and enhanced motor function, which was related to improving spinal cord motor neuron degeneration and reducing lipid peroxidation. In addition, GPX4 overexpression and chemical inhibitors of ferroptosis ameliorated SOD1^G93A^ induced cytotoxicity. The results show that ferroptosis plays a key role in motor neuron degeneration in ALS [266]. So far, most studies have focused on the contribution of ferroptosis to neural processes, but future studies should also focus on the therapeutic benefits of inhibiting ferroptosis in brain cells showing some characteristics of neurodegenerative diseases. We believe that ferroptosis is one of the most important forms of cell death in brain diseases. The in-depth study of ferroptosis will provide new opportunities for diagnosis and treatment intervention.

### Therapeutic implications

AD is a public health problem, but so far, only two types of drugs have been approved, including cholinesterase inhibitors and NMDA antagonists. Acetylcholinesterase inhibitors are divided into reversible, irreversible, and pseudo-reversible. By blocking the degradation of acetylcholine (Ach) by cholinesterase, the level of ACh in the synaptic cleft increases [267, 268]. Increasing cholinergic level by inhibiting acetylcholinesterase is considered to be one of the strategies to improve cognitive therapy for AD. Tacrine is the first cholinesterase inhibitor drug approved by FDA for the treatment of AD. Its effect is to increase acetylcholine in muscarinic neurons [269]. However, it withdrew from the market immediately after listing due to side effects such as hepatotoxicity. Later on, Donepezil, Rivastigmine, and Galantamine in use for the symptomatic treatment of AD [38, 268]. On the other hand, excessive activation of NMDA receptor (NMDAR) leads to an increase in the influx of Ca^2+^ levels, which promotes cell death and synaptic dysfunction. NMDAR antagonists can prevent excessive activation of NMDAR glutamate receptors, thereby preventing Ca^2+^ influx [270]. NMDAR is believed to play a leading role in the pathophysiology of AD. Excessive activation of NMDAR leads to abnormal Ca^2+^ signal levels and overstimulation of glutamate, which leads to excitotoxicity, synaptic dysfunction, neuronal cell death and cognitive decline. Some NMDAR uncompetitive antagonists have been developed and entered clinical trials. Memantine is the only drug approved for the treatment of moderate to severe AD [271, 272]. Disease modifying therapy (DMT) alters the progression of AD by exploring several pathophysiological mechanisms, which aim to improve cognitive function and alleviate symptoms such as depression or delusions. DMTs have been developed and advanced into clinical trials such as AN-1792 and AD active immunotherapy. Another class of enzymes that target α—secretase are considered therapeutic agents, which stimulate the cleavage of APP [273–275]. In addition to anti-amyloid drugs, inhibitors of tau aggregation are another promising DMT. Methylene blue, a blue dye that inhibits tau protein aggregation, has entered phase II clinical trials to treat mild to moderate AD [276]. Protein misfolding caused by mutations or environmental factors leads to toxic aggregation, and naturally, cells develop protein quality control (PQC) System, inhibiting protein misfolding before exerting its toxic effects. With age, this balance is altered and the misfolded shape overwhelms the PQC system, halting protein synthesis and increasing chaperone production. Molecular chaperones are therefore considered promising candidates for the treatment of neurodegenerative diseases [277, 278]. Recent studies have shown that natural compounds have neuroprotective effects and have therapeutic potential for AD. Nicotine was the first natural compound to enter clinical trials in AD, and then other compounds, such as vitamins C, E, and D, have also gained more attention and interest due to their protective effects on neuroinflammation and oxidative damage [279].

PD is the second most common neurodegenerative disease in the world, affecting 1% of people over 60 years old [280]. For decades, dopaminergic drugs have been considered as the main method to treat motor symptoms of PD. The combination of dopaminergic drugs monoamine oxidase (MAO) type B inhibitor, catechol-*O*-methyltransferase inhibitor (COMTI), anticholinergic drugs and other newly developed non dopaminergic drugs can better control motor symptoms or alleviate motor complications caused by levodopa [281]. Levodopa is the most effective drug for the treatment of PD, which combined with carbidopa or benserazide can prevent its peripheral metabolism and significantly reduce the risk of nausea [282]. Besides levodopa, anticholinergics, amantadine, MAOIs, COMTIs, dopamine agonists and istradefylline also available for the treatment of PD-related motor symptoms [283]. Anticholinergic drugs, such as trihexylphenyl and benzotropine, can antagonize the effect of postsynaptic muscarinic receptor acetylcholine on striatal interneurons. Amantadine is currently the main drug for the treatment of levodopa-related dyskinesias. In addition to its anti-glutamatergic effects, amantadine is also believed to stimulate the release of endogenous dopamine stores, prevent the reuptake of dopamine in the synaptic cleft, and has anticholinergic properties. MAO-B plays an indispensable role in DA metabolism in the brain. It can be used as an early monotherapy or in combination with levodpa. Selegiline is the first MAO-B inhibitor used in PD. It delays the need for levodopa by slowing the progression of PD [284, 285]. Safinamide is a reversible MAOI that reduces neuronal dopamine reuptake, blocks voltage dependent activated sodium channels and intracellular calcium entry, and thus reduces neuronal glutamate release [286]. COMT is an enzyme metabolizing levodopa. It is usually used in combination with levodopa and carbidopa. It has become a first-line drug for the treatment of PD motor fluctuation. COMTIs (entacapone, toccapone and apicapone) blocks the degradation of peripheral levodopa and toccapone which also blocks the central degradation of levodopa and dopamine increaseing the level of central levodopa and dopamine. Dopamine receptor agonists stimulate dopamine receptors. When introduced early in PD treatment, they delay levodopa related complications, such as motor fluctuations and dyskinesia. Non-ergot dopamine agonists commonly used in clinic include pramipexole, ropinirole, rotigotine and apomorphine [287]. In 2019, FDA approved istradefylline, an adenosine A2 receptor antagonist, as an adjuvant treatment of levodopa/carbidopa in patients with PD. The drug has a certain effect on patients with levodopa related motor fluctuations [288]. PD is a complex disease, and its pathogenesis involves many mechanisms, such as ROS, mitochondrial dysfunction, neuroinflammation, UPS, autophagy damage and other unknown mechanisms. Although great progress has been made in understanding the etiology of PD and the symptomatic treatment of PD related symptoms. However, there is currently no effective neuroprotective or DMT that can slow the progression of the disease.

HD is an autosomal dominant neurodegenerative disease characterized by progressive motor, behavioral and cognitive decline which caused by a pathogenic repeat expansion of the cytosine-adenine-guanine trinucleotide in exon 1 of the HTT gene on chromosome 4 [154]. There are no known drugs for HD and the treatment is only symptomatic. Tetrabenazine is the only drug with HD licensed indications for the treatment of choreiform movements, tetrabenazine reversibly inhibits vesicular monoamine transporter 2 in the CNS [289]. Silencing the expression of mutated Huntington genes using RNA interference (RNAi) or antisense oligonucleotide (ASOs) is effective in improving symptoms and pathology. However, there are some potential difficulties, including allele specificity, off-target effects, and delivery [290–292]. Glutamine residues in Huntington protein are crosslinked by transglutaminase, and its inhibitor cystamine has produced promising results in a mouse model of the disease [293]. Early human studies have shown the potential for survival of transplanted new neural tissues, although recent long-term follow-up studies are less encouraging and mutant Huntington protein has been found in transplanted tissues [294, 295]. Currently, therapeutic agents targeting HTT DNA to reduce Huntington protein can act by regulating gene transcription or directly modifying HTT gene. The standard method of DNA targeting is to use a combination of some form of specific DNA binding elements and effector elements such as nuclease, epigenetic regulator or transcription factor. At present, there are three main types of nucleases that can be designed for DNA targeting purposes: zinc finger nuclease, transcription activator like effector nuclease, and cas9 or other RNA guided bacterial nucleases [296]. At the post-transcriptional level, methods to regulate translation efficiency include RNAi, ASOs, and small molecule regulators of RNA processing. This triggers the cleavage of mHTT RNA, enhances degradation or translation inhibition, resulting in a decrease in the number of mutant HTT proteins produced. In addition to therapeutic approaches to reduce mHTT levels by targeting HTT DNA or RNA, there are potential small molecule huntingtin lowering technologies based on increasing the cellular clearance of mHTT protein. Misfolded and defective proteins, such as mHTT, are cleared from neurons via two major pathways: the UPS, which removes soluble and short-lived proteins by tagging them with ubiquitin and targeting them to the proteasome, leading to breakdown into single amino acids. Autophagy is a process in which larger cytoplasmic structures, such as aggregated proteins and damaged organelles, are degraded in double walled vesicular structures called autophagosomes and shuttled to lysosomes. Substantial evidence suggests that destabilization and / or inefficient degradation of mHTT and other misfolded proteins by the autophagy pathway leads to aggregation of toxic forms of mhtt within neurons, contributing to HD pathogenesis [297, 298]. Despite the discovery of the underlying genetic mutations in HD more than 20 years ago, we remain limited to treatments that address only the symptoms rather than the disease. With the understanding of pathogenesis and the identification of new potential therapeutic targets, we will make significant progress in the treatment of HD.

MS is a chronic inflammatory disease of the central nervous system that leads to demyelination and neurodegeneration. Although its etiology is still elusive, it is known that environmental factors and susceptibility genes are involved in the pathogenesis of the disease [299]. Aiming at the different pathogenic mechanisms of the progression of MS, compounds are being developed for immune system dysfunction, glial cells or neurons, metabolic abnormalities related to mitochondrial damage or different ion channels. In addition, trials of neuroprotective therapy aimed at preventing progress or partially reversing neurological dysfunction by repairing brain and spinal cord tissue are also currently ongoing. Ocrelizumab is a humanized monoclonal antibody that depletes B cells through antibody-dependent cell-mediated toxicity [300]. Fingolimod also showed some efficacy in the chronic EAE model, which is related to the ability to reduce pathology [301]. Studies have shown that mitochondria are potential therapeutic targets, and MitoQ, a specific inhibitor of mitochondrial ROS production, has protective properties in EAE. Because the level of inflammation is not affected by MitoQ, increasing the protection of mitochondria from ROS is sufficient to reduce axon damage [302]. Inhibition of Na^+^ channel and Ca^2+^-mediated activators is a reasonable therapeutic target, which can delay axon degeneration and permanent disability in patients with MS. In EAE, systemic administration of flucanamide or Na^+^ channel-blocking anticonvulsants (lamotrigine, phenytoin, carbamazepine) can reduce neurological dysfunction [303, 304]. The activation of ion channels ASIC1 and TRPM4 contributes to Na^+^ influx. Blocking these ion channels with amiloride or glibenclamide, respectively, may be a new method because they provide neuroprotection in EAE and reduce neurons and oligodendrocyte damage [305, 306]. AMPA/kainic glutamate receptor NBQX treatment can reduce neurological dysfunction in patients with EAE, increase the survival rate of oligodendrocytes, and reduced axon damage. The combined method of blocking AMPA/kainate and NMDA receptors may be an effective target for protecting glial and axons [307, 308]. Some evidence suggests that myelin and oligodendrocyte derived factors support axons, and their loss leads to axonal degeneration. Glial cell-derived neurotrophic factor (GDNF), insulin-like growth factor 1 and brain-derived neurotrophic factor are related to the nutritional support provided by oligodendrocytes for axons, which is predicted to be defective in multiple sclerosis. Therefore, these factors can be used as axon protective agents in treatment [309]. LINGO1 is a central nervous system specific membrane glycoprotein that inhibits oligodendrocyte differentiation and myelination. It is related to remyelination and axon repair failure in MS. Blocking this protein can effectively promote myelin regeneration [310]. The method suitable for MS treatment is still controversial. The future of MS treatment largely depends on a comprehensive understanding of the immune pathogenesis of MS.

ALS is a fatal progressive neurodegenerative disease characterized by permanent degeneration of upper and lower motor neurons. Many different genes and pathophysiological processes lead to this disease, but the exact cause is not clear. There is no known treatment for ALS, but there are two recognized treatments and the longest available one is riluzole. Since its approval in 1995, it is the only targeted therapy for ALS. Riluzole is an anti-glutamatergic drug. Its targeted excitotoxicity is considered to play a role in the pathophysiology of ALS [311, 312]. The second treatment to change the disease is edaravone, a powerful antioxidant that is reported to eliminate lipid peroxides and hydroxyl radicals. The mechanism of edaravone in ALS is as uncertain as that of riluzole. It is speculated that the drug alleviates the oxidative damage of neurons and adjacent glial cells at risk of degeneration in ALS [312]. The focus of anti-apoptosis is the mitochondrial damage of injured motor neurons and the abnormal calcium treatment leading to the apoptotic cascade. Two studies with preliminary reports on the use of Ursodeoxycholic acid and Tauroursodeoxycholic acid have moderately positive prospects [312, 313]. Studies have shown that the neuroinflammatory process related to reactive astrocytes and microglia plays an important role in ALS neurodegeneration [314, 315]. In a phase III study, the combination of masitinib and riluzole showed that the progression of ALS slowed down by 27%, showing great therapeutic potential [316]. Glutamate is the main regulator of excitotoxicity. In ALS, excitotoxicity comes from the excessive release of glutamate and the changes of postsynaptic glutamate receptor and glutamate transport. The main treatment for the pathophysiological pathway of excitotoxicity is the previously mentioned riluzole [317]. Oxidative stress may be one of the important factors in the pathogenesis of ALS. Targeting this pathophysiological pathway is edaravone approved for the second treatment of ALS [312]. In addition, Aeol is considered to be the most promising antioxidant for the treatment of ALS. It is a small molecule that catalyzes the consumption of reactive oxygen species and reactive nitrogen species [318]. Cellular protein aggregation is a known feature of ALS. SOD1 mutation leads to conformational instability, disorder and the formation of SOD1 protein aggregates [319, 320]. Preventing these cells from aggregating can improve the survival rate of motor neurons. Recently, macrophage migration inhibitory factor (MIF) has shown an inhibitory effect on toxic misfolded SOD1 amyloid aggregates. MIF changed the typical SOD1 amyloid aggregation pathway in vitro, but promoted the formation of disordered aggregation [321]. Another form of pathological aggregation of affected neurons in patients with amyotrophic lateral sclerosis is TDP-43, a major nuclear RNA binding protein. TDP-43 aggregation antagonist is an acridine derivative, [4,5-bis{(N-carboxy methyl imidazolium)methyl}acridine] dibromide. The results show that it separates the low complexity domain of adjacent continuous TDP-43 monomers, which destroys the formation of pathological aggregates [322]. Both drugs are considered as potential candidates for ALS treatment. The main aspect of the pathophysiology of ALS is the degeneration of neuronal tissue. The research of neurotrophic and neuroprotective therapy focuses on drugs that stimulate the repair of damaged neurons and promote the growth of new neurons. 7,8-dihydroxyflavone and other substances can improve the survival rate of affected neurons in ALS mouse model. It has a protective effect on mutant TDP-43 stress, showing a good potential for neuroprotective treatment of ALS [323]. Studies have shown that ASOs treatment can significantly delay disease progression in ALS rodent model with SOD1 mutation [324]. Another useful method is to transfer GDNF through viral vector AAV serotype 9. The strategy of AAV9-SOD1-shRNA decreased the incidence rate of SOD1 mice and SOD1 rats, and prolonged the survival time [325]. In short, scientists have been looking for an effective treatment for ALS.

## Conclusions and perspectives

During the development of normal neurons, PCD occurs in a space and time limited manner. Abnormal activation of PCD pathway is a common feature of neurodegenerative diseases, such as apoptosis, pyroptosis, autophagy dependent cell death, necroptosis and ferroptosis, resulting in the accidental loss of neuronal cells and functions. The molecular mechanisms underlying these distinct forms of cell death are not independent and recent evidence indicates that there are complex interplays among them, the crosstalk between these processes is the main cause of neuronal death [326]. Further study on the mechanism of these molecules will provide potentially important discoveries for crosstalk targeted therapy in neurodegenerative diseases. In this review, we compared the relationship between different types of cell death and neurodegenerative diseases in terms of induction factor, executor and pharmaceutical infection link to neurodegenerative diseases (Table 1). Meanwhile, we briefly described the cell death process of PCD and their role in promoting brain neurodegenerative diseases. We also discussed the interaction between different cell death signal cascades and disease pathogenesis, described therapeutic targets targeting key roles in cell death signal pathway, and finally reviewed the current treatment methods and promising methods of AD, PD, ALS, MS and HD. Generally, the mechanism of how to choose cell death is dependent on the cell type, stimulus, context and environment. It is particularly important to deeply study the mechanism of cell death and find a regulatory target with less side effects and good therapeutic effect.

**Table 1:** Comparison of different types of cell death

Type of cell death	Induction factor	Executioner	Morphological features	Biochemical halmarkers	Plasma membrane and nucleus	Chromatin	Other unique feature	Inflammatory reaction	Pharmacological inhibition
Apoptosis	Initiator caspases (caspase-2/8/9/10)	Executioner caspases (caspase-3/6/7)	Cellular shrinkage, dense cytoplasm, tightly packed organelles, apoptotic body formation	Activation of initiator and executioner caspases	Cell shrinkage, the plasma membrane with preserved integrity and nuclear compaction and fragmentation	Marked chromatin condensation	Apoptosome	No inflammatory reaction	Caspase-8 inhibitor, pan-caspase inhibitors
Pyroptosis	Inflammatory caspase	GSDMD	Rupture of plasma membrane, chromatin condensation, blebbing of the cell membrane	Inflammatory caspase activation (caspase-1 and caspase-11/4/5)	Rapidly plasma-membrane rupture, cell swelling	DNA fragmentation without nuclear condensation	Inflammasome	Lytic inflammatory reaction	Inflammasome inhibitor (MCC950, Bay 1 1–7082, JC-124), pan-caspase inhibitors, caspase-1 inhibitor
Autophagy-dependent cell death	Formation of PAS (pro-autophagosome)	Lysosomes	Focal plasma membrane rupture, autophagosome with a double-membrane, mild moderate chromatin condensation	Dependency on autophagy machinery	Focal plasma membrane rupture	Mild moderate chromatin condensation	Autophagosome	No inflammatory reaction	Autosis inhibitors
Necroptosis	TNT, TN'FRl, FAS, TRAILR1	MLKL	Cellular swelling, dense cytoplasm, tightly packed organelles	RIPK activation	Cell swelling; plasma membrane rupture; organelle swelling	Mild moderate chromatin condensation	Necrosomes	Inflammatory reaction	RIP1 inhibitors lnecrostaiin-1). RIP3 inhibitors (GSKS43. GSK-872).MLKL inhibitor
Ferroptosis	Erastin, (1S, 3R)-RSL3	Lipid peroxidation	Iron and reactive oxygen species (ROS) dependent, decreased or vanishing mitochondrial crista, a condensed mitochondrial membrane, and a ruptured outer mitochondrial membrane	Glutathione Peroxidase inactivation, iron-dependent ROS accumulation	Cell volume shrinkage and increased mitochondrial membrane density	Chromatin fragmentation	Accumulation of intracellular ROS	Inflammatory reaction	Iron chelators, (deferoxamine, DFO), lipid peroxidation inhibitors, vitamin E, ferrostatin-1 (Fer-1), and liproxstatin-1 (Lip-1)

## Data Availability

Not applicable.

## Abbreviations

Aβ: Amyloid β-protein
ACD: Accidental cell death
Ach: Acetylcholine
ACSL4: Acyl-CoA synthetase long-chain family member 4
AD: Alzheimer’s disease
ALS: Amyotrophic lateral sclerosis
APP: Amyloid precursor protein
APAF1: Apoptotic protease-activating factor 1
ASOs: Antisense oligonucleotide
ATG: Autophagy-related genes
BH: BCL-2 homology
CARD: C-terminal caspase recruitment domain
cIAP1: Cellular inhibitor of apoptosis protein 1
CMA: Chaperone mediated autophagy
CNS: Central nervous system
COMTI: Catechol-*O*-methyltransferase inhibitor
CQ: Chloroquine
CZ: Cuprizone
DFO: Deferoxamine
DMT: Disease modifying therapy
DRs: Death receptors
EAE: Experimental allergic encephalomyelitis
Fer-1: Ferrostatin-1
FSP1: Ferroptosis suppressor protein 1
FTH1: Ferritin heavy chain 1
GDNF: Glial cell-derived neurotrophic factor
GPX4: Glutathione peroxidase 4
GSDMD: Gasdermin-D
GSH: Glutathione
HD: Huntington’s disease
HSC70: Heat shock cognate 70 kDa protein
HTT: Huntingtin
LAMP2A: Lysosomal associated membrane protein 2A
LC3-PE: Light chain 3-phosphatidylethanolamine
LIR: LC3-interacting region
MAO: Monoamine oxidase
MAPK: Mitogen-activated protein kinase
mHTT: Mutant huntingtin
MIF: Migration inhibitory factor
MLKL: Mixed-lineage kinase domain-like protein
MOMP: Mitochondrial outer membrane permeabilization
MPTP: 1-Methyl-4-phenyl-1,2,3,6-tetrahydropyridine
MS: Multiple sclerosis
NCCD: Nomenclature committee on cell death
NCOA4: Nuclear receptor coactivator 4
Nec-1: Necrostatin-1
NF-κB: Nuclear factor-κB
NLR: NOD-like receptor
NLR: NOD-like receptor
NMDA: *N*-Methyl d-aspartate
NMDAR: NMDA receptor
OPTN: Optineurin
PCD: Programmed cell death
PD: Parkinson’s disease
PQC: Protein quality control
PS: Presenilins
PtdSer: Phosphatidylserine
PUFAs: Polyunsaturated fatty acids
RCD: Regulated cell death
RNAi: RNA interference
RIPK1: Receptor-interacting serine/threonine protein kinase 1
RIPK3: Receptor-interacting serine/threonine protein kinase 3
ROS: Reactive oxygen species
RSL3: RAS selective small molecule lethal 3
SNpc: Substantia nigra pars compacta
TNF: Tumor necrosis factor
TNFR1: Tumor necrosis factor receptor-1
TRADD: TNFR1-associated death domain
TRAF2: TNFR-associated factor 2
TUNEL: Terminal deoxynucleotidyl transferase-mediated dUTP nick-end labelling
UBD: Ubiquitin-binding domain
UPS: Ubiquitin–proteasome system
3-NP: 3-Nitropropionic acid
